# IL-21 enhances the cytotoxicity of intratumoral CD8^+^ T cells, improving radiation efficacy

**DOI:** 10.1172/jci.insight.190531

**Published:** 2026-01-08

**Authors:** Xin-yang Li, Xue-qi Xie, Bao-chao Wei, Xiao-zheng Sun, Min-xin Chen, Ru-fei Liu, Qing-xu Tao, Yi-heng Huang, Qian Wang, Shuang-shuang Ma, Ling Wei, Rong Xiao, Zhao-yun Liu, Jin-ming Yu, Meng Wu, Dawei Chen

**Affiliations:** 1 Department of Radiation Oncology and Shandong Provincial Key Laboratory of Precision Oncology, Shandong Cancer Hospital and Institute Shandong First Medical University and Shandong Academy of Medical Sciences, Jinan, Shandong, China.; 2 Cancer Center, Shandong University, Jinan, Shandong, China.

**Keywords:** Oncology, Therapeutics, Cancer immunotherapy, Radiation therapy, T cells

## Abstract

Radiotherapy is a critical modality in cancer treatment, not only to eradicate cancer cells but also to trigger antitumor immunity. IL-21, an immunomodulatory cytokine with potential in cancer therapy, has unexplored synergy with radiotherapy. Our study, leveraging human cancer databases and tissue microarrays, identified a positive correlation between IL-21 and radiotherapy outcomes, particularly in tumor microenvironment (TME) activation. In mouse tumor models, IL-21 combined with radiation significantly enhanced the TME, boosting CD8^+^ T cell activation and function, reducing tumor burden, and extending survival. Single-cell transcriptome sequencing revealed that the combination of IL-21 and radiation increased the cytotoxicity of effector and memory CD8^+^ T cells and prevented their exhaustion. These effects were further validated in humanized mice, where IL-21 combined with radiation reduced A549 tumor growth and enhanced CD8^+^ T cell function. Post-neoadjuvant radiotherapy samples from patients with esophageal cancer showed a positive correlation between IL-21 levels and CD8^+^ T cell infiltration. Our findings suggest that IL-21 is a promising adjuvant to radiotherapy, potentially improving treatment efficacy through TME enhancement. This study provides a foundation for future clinical exploration of IL-21 for enhancing radiotherapy.

## Abstract 2

IL-21 combined with radiation boosted anti-tumor efficacy via increased infiltration and boosted function of intratumoral CD8^+^ T cells, providing experimental evidence for clinical employment of IL-21 as an immunomodulator combined with radiotherapy.

## Graphical abstract

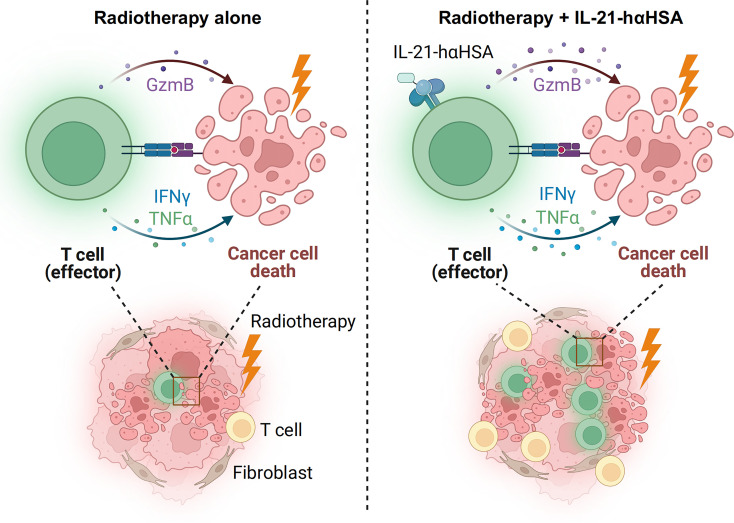

## Introduction

Radiotherapy is a pivotal treatment modality in oncology and is utilized to manage a variety of cancers by inducing direct tumor cell death and invoking systematic antitumor immune responses (1). Despite its widespread application, the development of resistance to radiotherapy remains a critical issue that often leads to treatment failure and disease recurrence. This resistance can arise through multiple mechanisms, including enhanced DNA repair (2), cell cycle changes (3), activation of survival pathways, and an immunosuppressive tumor microenvironment (TME) (3, 4), which collectively enable tumor cells to withstand the cytotoxic effects of radiation and evade immune surveillance. Therefore, overcoming radiotherapy resistance is a significant challenge for cancer treatment.

Although the immunomodulatory effects and possible mechanisms of hypofractionated or extreme hypofractionated radiotherapy and conventional radiotherapy are different in certain aspects (5, 6), enhancing antitumor immunity is a well-acknowledged, promising approach to improve the efficacy of radiotherapy. The TME is often immunosuppressive, limiting the effectiveness of immune responses against tumor cells (7). A key component of this immune response is cytotoxic CD8^+^ T cells, which can directly kill cancer cells through the release of cytotoxic granules and engagement of death receptors on tumor cells (8). As reported in both clinical and preclinical research, radiotherapy, especially hypofractionated radiotherapy, could lead to significant CD8^+^ T cell enrichment in the TME (9, 10), and the infiltration and function of CD8^+^ T cells within the TME are crucial for effective immunosurveillance and response to radiotherapy. The combination of radiotherapy and immune checkpoint inhibitors, such as PD-1, PD-L1, and CTLA-4, has achieved clinical success in various types of cancers (11, 12). DC-based immunotherapy, which promotes the cross-priming of CD8^+^ T cells to tumor antigens after radiotherapy and increases the tumor infiltration of CD8^+^ T cells (13), has exhibited promising clinical benefits in a phase I trial in patients with hepatocellular carcinoma treated with single-fraction radiotherapy followed by DC injection (14). In contrast, cytokines, which can enhance the proliferation and effector function of CD8^+^ T cells (15), have emerged as potential immunomodulatory partners for radiotherapy. Therefore, targeting CD8^+^ T cells within the TME to enhance their cytotoxicity represents a promising strategy for overcoming radiotherapy resistance, especially for hypofractionated radiotherapy.

The IL-2 family of cytokines, including IL-2, IL-12, IL-15, and IL-21, play a pivotal role in regulating immune responses, especially in the activation and proliferation of T cells (16). Recently, IL-2 has been used as a therapeutic agent in cancer treatment to enhance the proliferation of T cells and NK cells, thereby boosting the immune response against cancer (17). However, its systemic administration is associated with significant toxicity (18), which has led to the exploration of other IL-2 family members with potentially more favorable safety profiles. IL-21, another member of the IL-2 family, has emerged as a promising therapeutic agent for cancer. It is primarily produced by activated CD4^+^ T cells and prevents T cell differentiation and preserves a naive-like phenotype during ex vivo expansion (19). Within the TME, IL-21 derived from CD4^+^ T cells maintains the effector function of tumor-infiltrating CD8^+^ T cells and is regarded as an important target to augment antitumor efficacy (20). In addition, IL-21 has been used to enhance the antitumor effects of adoptive immunotherapy by improving the propagation of CAR-T cells (21). The antitumor effectiveness of IL-21 has been validated in various preclinical models, including renal cell carcinoma, melanoma, pancreatic cancer, and breast cancer (22–25). Clinical trials have further confirmed the efficacy of IL-21, both as a standalone treatment and in combination with other therapeutic modalities (such as immune checkpoint inhibitors, oncolytic virus, CAR-T/CAR-NK therapies, and cytokines) for the management of human solid tumors (19). However, whether the addition of IL-21 benefits radiotherapy remains unclear.

Our study found that IL-21 is positively correlated with radiotherapy prognosis and a favorable TME, and that exogenous administration of IL-21 could synergistically enhance radiation efficacy with tolerable toxicity. IL-21 combined with radiation significantly improves the TME, especially by increasing infiltration and augmenting the cytotoxicity of effector CD8^+^ T cells, and IL-21 could promote effector function and curb the exhaustion of CD8^+^ T cells via direct action on CD8^+^ T cells after radiation. Overall, our findings provide a comprehensive understanding of the role of IL-21 in enhancing the cytotoxicity of intratumoral CD8^+^ T cells and its potential to improve the efficacy of radiation therapy, thereby offering potentially new insights into the development of more effective cancer treatments.

## Results

### High expression of IL-21 is positively correlated with the prognosis of radiotherapy in various types of cancer.

**Figure 1:**
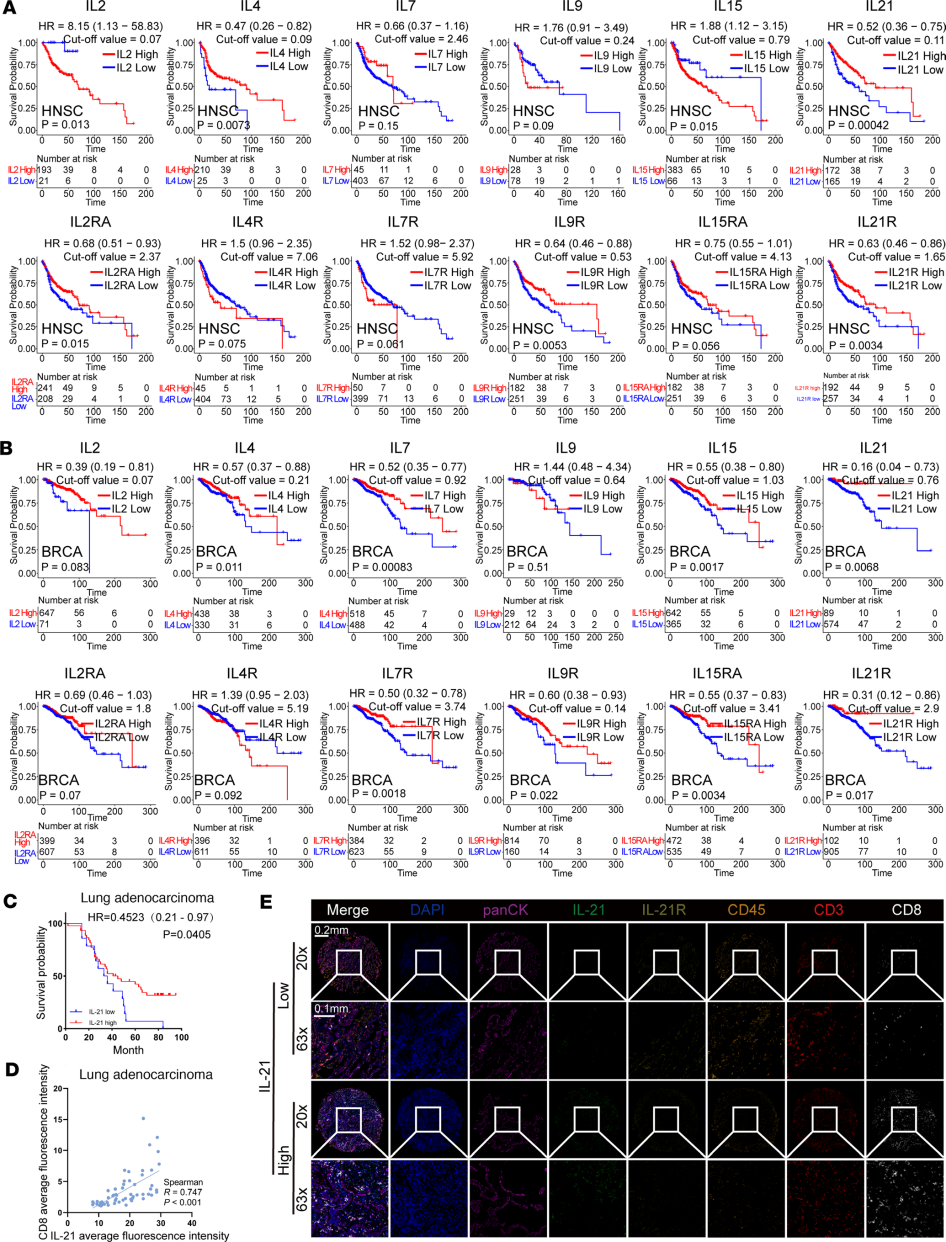
Expression of *IL21* in the TME is correlated with favorable prognosis of radiotherapy and increased CD8^+^ T cell infiltration. (**A**) Kaplan-Meier analysis of the overall survival of radiotherapy-treated patients with HNSC with high or low expression of *IL2*, *IL4*, *IL7*, *IL9*, *IL15*, and *IL21* and corresponding receptors (*IL2RA*, *IL4R*, *IL7R*, *IL9R*, *IL15RA*, and *IL21R*) from TCGA database. (**B**) Kaplan-Meier analysis of the overall survival of radiotherapy-treated patients with breast cancer and high or low expression of *IL2*, *IL4*, *IL7*, *IL9*, *IL15*, and *IL21* and corresponding receptors (*IL2RA*, *IL4R*, *IL7R*, *IL9R*, *IL15RA*, and *IL21R*) from TCGA database. (**C**) Kaplan-Meier analysis of overall survival of patients with lung adenocarcinoma with high or low *IL21* expression. (**D**) Correlation analysis of expression of IL-21 and CD8^+^ T cells in lung adenocarcinoma samples. (**E**) Representative data of IL-21, IL-21R, CD45, CD3, CD8, and panCK expression in lung adenocarcinoma samples. Scale bars: 0.1 mm and 0.2 mm.

Cytokines of the IL-2 family are known to exert significant effects on the initiation, progression, and therapeutic response of tumors. To elucidate the prognostic relevance of IL-2 family members, the Kaplan-Meier plotter database was utilized to assess the correlation between the expression levels of *IL2*, *IL4*, *IL7*, *IL9*, *IL15*, and *IL21* with overall survival across a broad spectrum of cancer types. However, in the datasets collected in the Kaplan-Meier plotter database, the expression of IL-2 family members, especially *IL2*, *IL4*, *IL9*, and *IL21*, was too low for meaningful statistical analysis. We therefore investigated the correlation between the expression of the receptors of IL-2 family, *IL2RA*, *IL4R*, *IL7R*, *IL9R*, *IL15RA*, and *IL21R*, and prognosis of various cancer types. The results indicated that among these cytokine receptors, only the expression level of *IL21R* was positively correlated with the prognosis of multiple tumor types, such as cervical squamous cell carcinoma, lung adenocarcinoma, ovarian cancer, pheochromocytoma and paraganglioma, rectum adenocarcinoma, and uterine corpus endometrial carcinoma (Supplemental Figure 1, A–F; supplemental material available online with this article; https://doi.org/10.1172/jci.insight.190531DS1). To further investigate the correlation between IL-2 family members and the prognosis of radiotherapy, The Cancer Genome Atlas (TCGA) database was utilized to perform correlation analysis of the expression of *IL2*, *IL4*, *IL7*, *IL9*, *IL15*, and *IL21* and their corresponding receptors with radiotherapy prognosis. To conduct a valid statistical analysis, raw data were downloaded from the latest TCGA database (version v43.0, released on May 7, 2025). Patients who received radiotherapy were included, and patients with undetectable mRNA expression of IL-2 family members and corresponding receptors were excluded for respective analysis. As for the head and neck squamous cell carcinoma (HNSC) cohort, since HPV status significantly affects radiotherapy prognosis (26), we integrated the previously reported HPV status of patients with HNSC (27) with the radiotherapy-treated HNSC cohort we obtained from TCGA dataset for analysis. Subsequently, the correlation between the expression of relevant genes and prognosis was analyzed in various cancer types with the screened data according to the above inclusion and exclusion criteria using R scripts (https://github.com/susiexueqi/TGCA_HNSC; commit ID 71b6545). The results showed that the expression of *IL21* and its receptor *IL21R* was consistently positively correlated with radiotherapy prognosis in the HNSC and colon adenocarcinoma, breast cancer, and skin cutaneous melanoma cohorts (Figure 1, A and B, and Supplemental Figure 2). Furthermore, we observed that the expression of *IL21* and *IL21R* was positively correlated with radiotherapy prognosis in both HPV^+^ and HPV^–^ patients with HNSC (Supplemental Figure 3A). These findings highlight the distinct role of *IL21* in modulating cancer progression and patient survival. Previous research has established that the infiltration level of immune cells, especially T cells in the TME, is commonly positively correlated with the prognosis of patients with cancer (28). Therefore, multiplex immunofluorescence was further employed to detect and analyze the expression and correlation of IL-21, IL-21R, CD45, CD3, and CD8 in the TME of lung adenocarcinoma. Consistent with the aforementioned database analysis, we found that high IL-21 expression predicted a better prognosis (Figure 1C). Additionally, we observed that high expression of IL-21 was positively correlated with increased infiltration of CD45^+^ immune cells, CD3^+^ T cells, and CD8^+^ T cells, and high expression of IL-21R was also positively associated with greater enrichment of CD3^+^ T cells (Figure 1, D and E, and Supplemental Figure 3B). In summary, these results suggest that high levels of IL-21 may improve the TME and enhance the efficacy of radiotherapy.

### Exogenous administration of IL-21 synergistically enhances radiation efficacy.

Although IL-21 has antitumor effects, its safety and efficacy are the main factors that limit clinical outcomes. It has been reported that compared with IL-21, an engineered fusion protein combining IL-21 with an anti–human serum albumin nanobody (IL-21-αHSA) exhibits better bioavailability and stability with an extended half-life and has demonstrated antitumor effects in murine tumor models (29). Therefore, IL-21-αHSA was used in our study and was termed IL-21 in the following experiments. The antitumor effects and drug safety of exogenous administration of IL-21 were explored. A subcutaneous tumor model derived from the murine colorectal carcinoma cell line MC38 was established, and varying doses of IL-21 were administered intraperitoneally. The results showed that 1.0–3.0 mg/kg IL-21 significantly inhibited MC38 tumor growth (Supplemental Figure 4A) without apparent drug toxicity observed, since there was no significant weight loss in the mice (Supplemental Figure 4B) and no significant changes were found in vital organs, such as the heart, brain, lung, thymus, liver, kidney, spleen, and lymph nodes (Supplemental Figure 4C). Therefore, 1.0 mg/kg for subsequent in vivo experiments was utilized in the following experiments.

**Figure 2:**
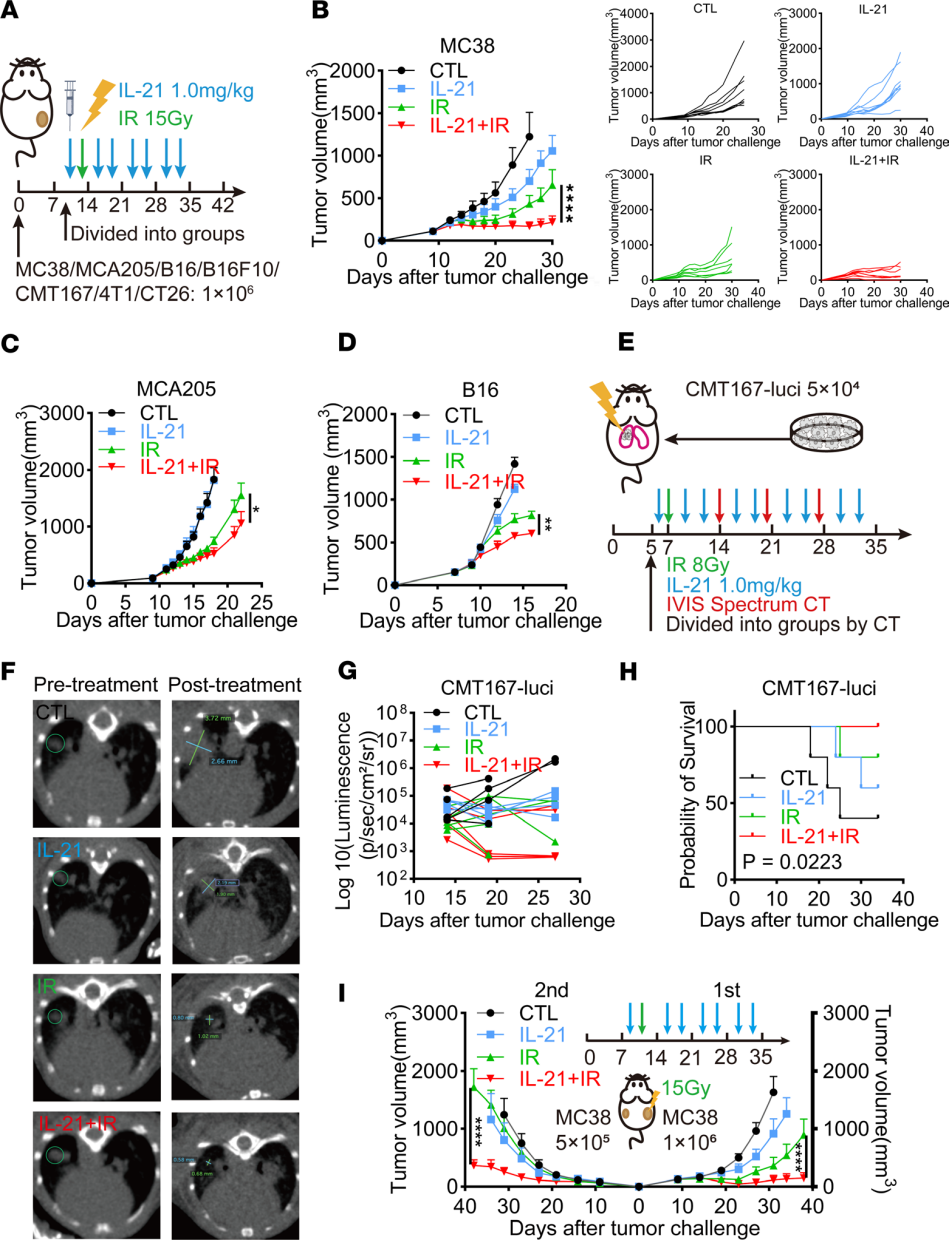
Exogenous administration of IL-21 synergistically enhances the antitumor effects of radiation. (**A**) Schematic diagram of IL-21 combined with radiation treatment for subcutaneous tumor models. (**B**–**D**) Tumor growth of MC38, MCA205, and B16 tumors in immunocompetent C57BL/6J mice treated with radiation with or without IL-21. (**E**) Schematic diagram of lung orthotopic tumor implantation and treatment process. (**F**) Representative data of CT images of lung orthotopic tumors. (**G**) Quantified total flux of luminescence of lung orthotopic tumor–bearing mice treated as in **E**. (**H**) Survival curves of lung orthotopic tumor–bearing mice. (**I**) Tumor growth of bilateral MC38 tumors treated with radiation of tumor on the right flank with or without systematic administration of IL-21. Data are shown as mean ± SEM (*n* = 5–8 per group). Statistical analysis was performed using a mixed-effects model with Šidák’s multiple-comparison test (**B**), 2-way ANOVA with Šidák’s multiple-comparison test (**C**, **D**, and **I**), and log-rank (Mantel-Cox) test (**H**). *P < 0.05, **P < 0.01, ****P < 0.0001.

To further investigate whether exogenous administration of IL-21 could synergistically enhance the efficacy of radiation treatment, multiple murine tumor cell lines (including murine colorectal carcinoma cell lines MC38 and CT26, murine fibrosarcoma cell line MCA205, murine melanoma cell lines B16 and B16F10, murine lung adenocarcinoma cell line CMT167, and murine breast cancer cell line 4T1) were used to establish subcutaneous tumor models in immunocompetent mice, and exogenous IL-21 was administered concurrently with a single fraction of radiation (Figure 2A). Tumor growth curves revealed that although IL-21 alone did not markedly inhibit tumor growth in most tumor models, the combination of IL-21 and radiation showed synergistic antitumor effects in tumors of various immunogenicities (Figure 2, B–D, and Supplemental Figure 4, D–G). To further validate the role of IL-21 in enhancing radiation efficacy, an orthotopic lung tumor model in immunocompetent mice was established using luciferase-labeled CMT167 cells (Figure 2E and Supplemental Figure 4H). The combination of IL-21 and precise radiotherapy led to significant tumor attenuation, as evidenced by CT imaging and bioluminescence, and also dramatically extended the survival of tumor-bearing mice (Figure 2, F–H). According to previous reports, IL-21 primarily acts on various immune cells to exert its immunomodulatory effects. It is well known that radiotherapy can promote irradiated tumor regression as well as activate systematic antitumor immune responses, leading to abscopal effects (1). To further explore whether exogenous administration of IL-21 could enhance the abscopal effects of radiation, bilateral MC38 tumor models were established in immunocompetent mice, and 1 fraction of 15 Gy was administered to each tumor along with systemic administration of IL-21 (Figure 2I). The results showed that the addition of IL-21 notably inhibited the growth of the irradiated tumor and strikingly enhanced the abscopal effect, leading to the regression of the abscopal tumors (Figure 2I). This also indicated that IL-21 could enhance radiation-mediated antitumor immune responses.

In summary, these findings indicate that exogenous IL-21 could synergistically enhance the efficacy of radiation, which may be attributed to boosted antitumor immunity.

### Exogenous administration of IL-21 combined with radiation improves the TME.

**Figure 3:**
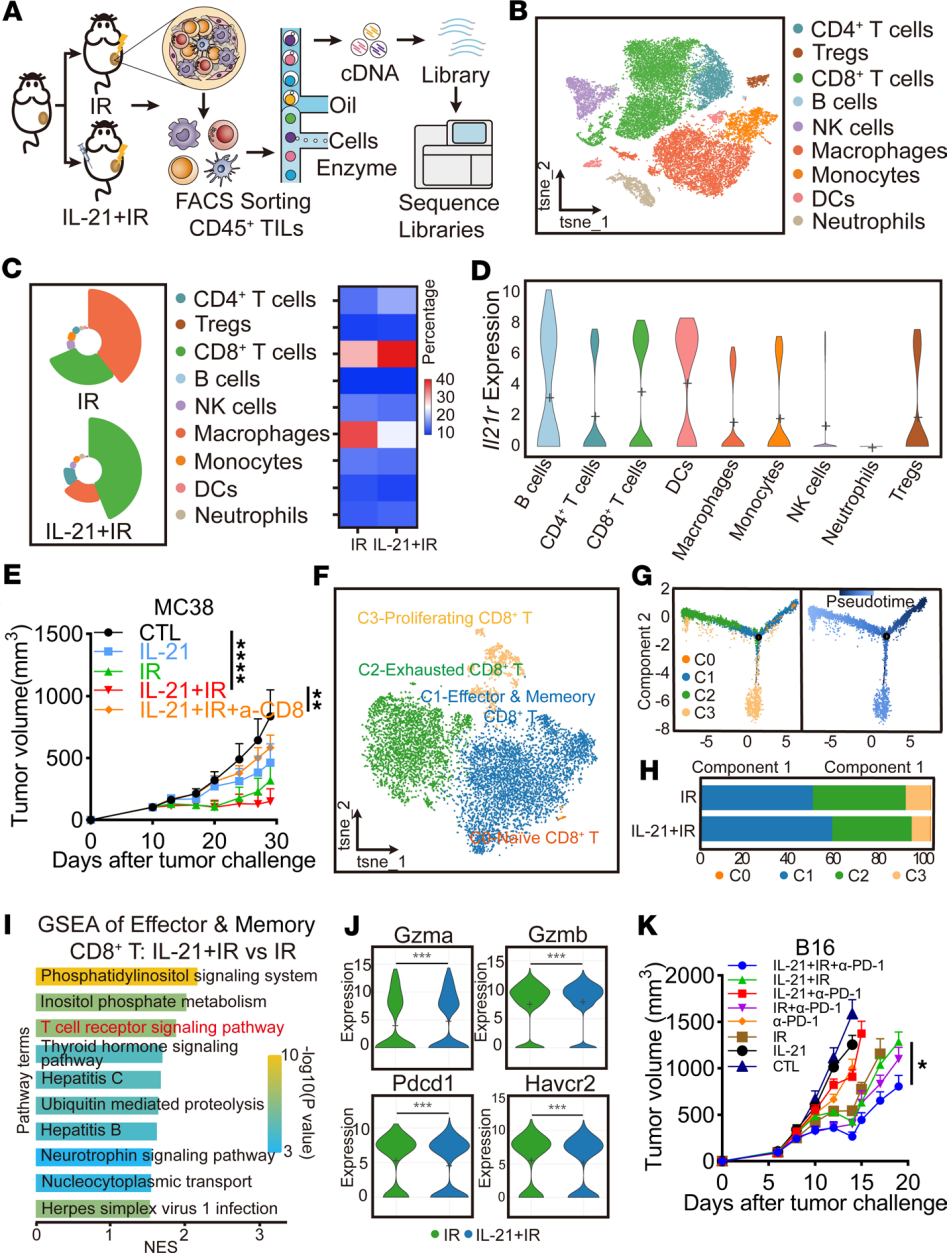
IL-21 combined with radiation reshapes the TME and increases the infiltration of CD8^+^ T cells. (**A**) Schematic diagram for scRNA-Seq of CD45^+^ immune cells sorted from MC38 tumors of IR group and IL-21+IR group 9–10 days after radiation. (**B**) t-SNE plot showing sorted CD45^+^ immune cells. (**C**) Nightingale rose chart and heatmap showing the proportion of different subtypes of CD45^+^ immune cells. (**D**) Violin plots showing *Il21r* expression in the different subtypes of CD45^+^ immune cells. (**E**) Tumor growth of MC38 tumors subjected to radiation and/or IL-21 with or without anti-CD8 depletion antibody. (**F**) t-SNE plot showing the subtypes of CD8^+^ T cells. (**G**) Pseudo-time analysis of the subtypes of CD8^+^ T cells. (**H**) Bar plot showing the proportion of different subtypes of CD8^+^ T cells. (**I**) GSEA of upregulated genes for effector and memory CD8^+^ T cells (C1) in IL-21+IR group versus IR group. (**J**) Violin plots showing the expression levels of *Gzma*, *Gzmb*, *Pdcd1*, and *Havcr2* of effector and memory CD8^+^ T cells of IR and IL-21+IR group from scRNA-Seq data. (**K**) Tumor growth of B16 tumors treated with radiation and/or IL-21 with or without anti–PD-1. Data shown as mean ± SEM in **E** (*n* = 5 per group) and **K** (*n* = 7 per group). Statistical analysis was carried out using 2-way ANOVA with Tukey’s multiple-comparison test (**E** and **K**). For panel **J**, differences between the 2 groups were assessed using the pairwise.wilcox.test() function in clusterProfiler. *P < 0.05; **P < 0.01; ***P < 0.001; ****P < 0.0001.

To further validate that the synergistic effects of exogenous administration of IL-21 in enhancing radiation efficacy were mediated by augmentation of antitumor immune responses, CD45^+^ tumor-infiltrating lymphocytes from MC38 tumors treated with radiation (IR group) or IL-21 combined with radiation (IL-21+IR group) were isolated 9 days after radiation followed by single-cell RNA-Seq (scRNA-Seq) (Figure 3A). After quality control, 27,818 CD45^+^ immune cells were collected and grouped into 9 cell types and 16 clusters (Figure 3B and Supplemental Figure 5, A and B). According to the marker genes, clusters 0, 2, 8, and 10 were identified as CD8^+^ T cells; clusters 1, 4, 12, 13, and 15 as macrophages and monocytes; clusters 3 and 14 as CD4^+^ T cells and B cells; and clusters 6, 9, 5, and 11 as neutrophils, DCs, NK cells, and Tregs, respectively (Supplemental Figure 5C). The abundance of different cell types was thoroughly analyzed, and the results illustrated that CD8^+^ T cells and macrophages exhibited the most pronounced changes in the IL-21+IR group compared with the IR group, with increased CD8^+^ T cell enrichment and decreased macrophage enrichment in the IL-21+IR group (Figure 3C). This indicated that CD8^+^ T cells and macrophages may be key contributors to the synergistic effects of IL-21 and radiation therapy. It has been reported that IL-21R is primarily expressed in various immune cells, including CD4^+^ T cells, CD8^+^ T cells, B cells, NK cells, DCs, macrophages, and mast cells (19), whereas the primary target immune cells of IL-21 are CD8^+^ T cells and NK cells in cancer immunotherapy (30). To rule out the key cell type in IL-21 enhancing radiation efficacy, the expression of *Il21r* in all immune cells was reanalyzed, and higher expression levels of *Il21r* were detected in CD8^+^ T cells than in macrophages (Figure 3D and Supplemental Figure 5D). Although the expression level of *Il21r* in CD8^+^ T cells was slightly lower than that in DCs, the proportion of DCs was unaltered in the IL-21+IR group subset (Figure 3, C and D, and Supplemental Figure 5D). Additionally, gene set enrichment analysis (GSEA) of the 2 groups using the Gene Ontology (GO) database and the Kyoto Encyclopedia of Genes and Genomes (KEGG) database revealed that T cell activation and T cell receptor signaling pathways were significantly enriched in the IL-21+IR group (Supplemental Figure 5, E and F). These analyses suggested that CD8^+^ T cells might be the predominant contributor. This hypothesis was validated by in vivo experiments, where the antitumor effects of the combination of IL-21 and radiation were completely abolished by depletion of CD8^+^ T cells (Figure 3E). Taken together, these findings demonstrated that CD8^+^ T cells are pivotal for the synergistic effects of IL-21 and radiation treatment.

A detailed analysis of CD8^+^ T cell subsets within the TME was conducted. A total of 9,832 CD8^+^ T cells were grouped into 4 clusters (Figure 3F and Supplemental Figure 5G). Cluster 0 was identified as naive CD8^+^ T cells, characterized by high expression of *Ccr7*, *Lef1*, and *Tcf7*. Cluster 1 cells were identified as effector and memory CD8^+^ T cells for marked expression of *Cxcr3*, *Gzmk*, *Gzma*, and *Ccl5*. Exhaustion-related genes (*Pdcd1*, *Havcr2*, *Tigit*, and *Tox*) were enriched in cluster 2, defined as exhausted CD8^+^ T cells, and cluster 3 was identified as proliferating CD8^+^ T cells due to the high expression of *Mki67* and *Top2a* (Supplemental Figure 5, H and I). The trajectory of CD8^+^ T cells (clusters 0, 1, 2, and 3) suggested a possible path for CD8^+^ T cell exhaustion, with naive CD8^+^ T cells (cluster 0) positioned at the root site, followed by effector and memory CD8^+^ T cells (cluster 1) and exhausted CD8^+^ T cells (cluster 2); proliferating CD8^+^ T cells (cluster 3) constituted another branch (Figure 3G). Further analysis of the proportion of each subset of CD8^+^ T cells showed notably increased effector and memory CD8^+^ T cells and reduced exhausted CD8^+^ T cell enrichment in the IL-21+IR group compared with the IR group (Figure 3H). GSEA of effector and memory CD8^+^ T cells revealed that the upregulated gene sets in the IL-21+IR group were mainly enriched in the T cell receptor signaling pathway (Figure 3I). In contrast, no immune-related pathway was significantly activated in the other 3 CD8^+^ T cell subgroups (Supplemental Figure 5, J–L). These results suggest that the addition of IL-21 exerted strong effects on T cell activation function for effector and memory CD8^+^ T cells.

Considering that CD8^+^ T cell activation is usually associated with the release of cytotoxic cytokines, the expression of representative cytokines *Ifng*, *Tnf*, *Gzma*, and *Gzmb* was analyzed in each subgroup. Consistently, the expression of *Gzma* and *Gzmb* of effector and memory CD8^+^ T cells was significantly higher in the IL-21+IR group than in the IR group (Figure 3J). IL-21 combined with IR could significantly reduce the expression of *Pdcd1* and *Havcr2* in exhausted T cells (Figure 3J). Interestingly, *Ifng* was highly expressed in the exhausted CD8^+^ T cell subset, indicating that exhausted CD8^+^ T cells still maintained cytotoxic functions (Supplemental Figure 5I). Also, these transcriptome data suggest that IL-21 boosted the effector and cytotoxic function of CD8^+^ T cells after radiation. According to previous research, the fusion antibody of PD-1 and IL-21 can enhance antitumor efficacy by reversing CD8^+^ T cell exhaustion (31). In line with this, our study found that the addition of anti–PD-1 dramatically enhanced the antitumor effects of IR combined with IL-21 in both B16 and CMT167 tumor models (Figure 3K and Supplemental Figure 5M). Furthermore, IL-21 significantly boosted the abscopal effects induced by anti–PD-1 combined with radiation in B16 tumor models (Supplemental Figure 5N). Altogether, these observations demonstrated that exogenous administration of IL-21 boosted the infiltration and cytotoxicity of CD8^+^ T cells in the TME after radiation, improving therapeutic efficacy.

### IL-21+IR enhances cytotoxicity of CD8^+^ T cells in the TME.

**Figure 4:**
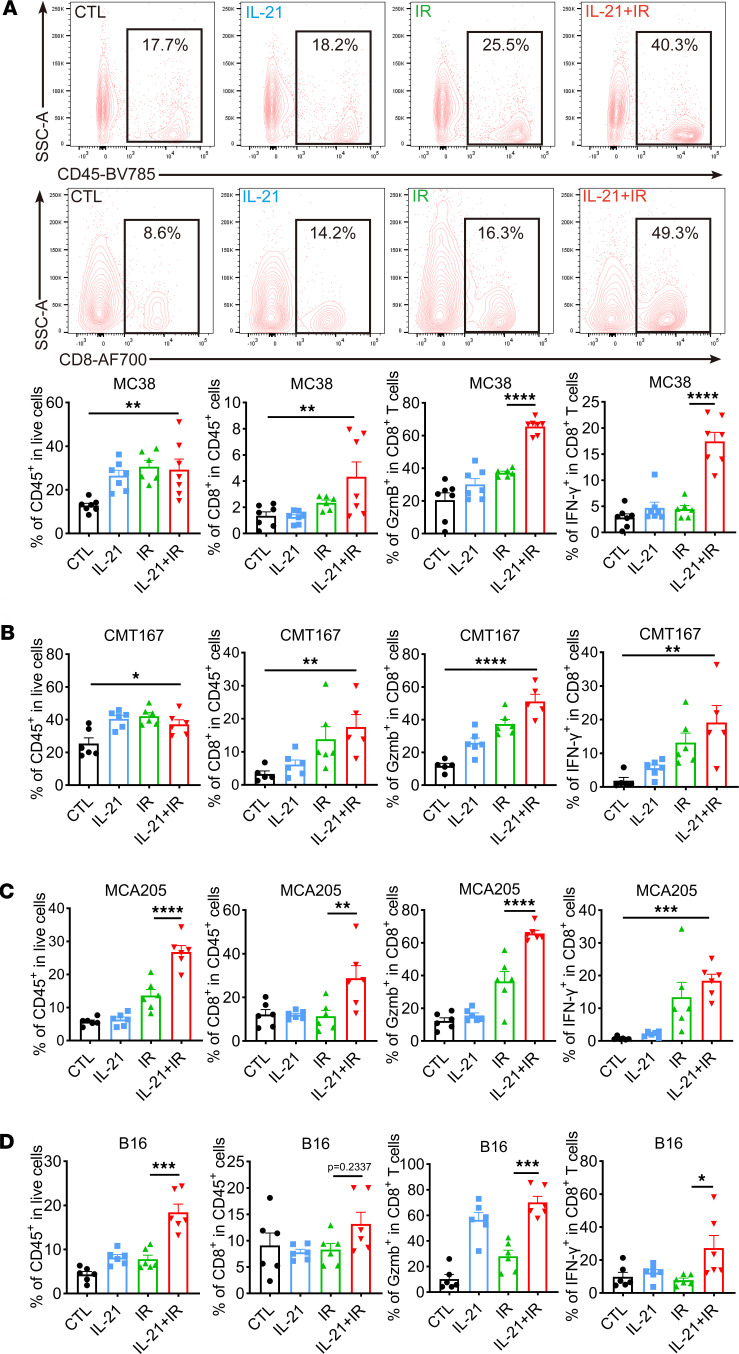
IL-21 combined with radiation enhances the cytotoxicity of CD8^+^ T cells in the TME. (**A**) Representative data and quantitative analysis of CD45^+^ cells, CD8^+^ T cells, Gzmb^+^CD8^+^ T cells, and IFN-γ^+^CD8^+^ T cells from MC38 tumors subjected to radiation with or without IL-21. (**B**–**D**) Quantitative analysis of CD45^+^ cells and CD8^+^ T cells from CMT167, MCA205, and B16 tumors subjected to radiation with or without IL-21. Data are shown as mean ± SEM (*n* = 5–8 per group). Statistical analysis was carried out using 1-way ANOVA followed by Tukey’s multiple-comparison test (**A**–**D**). *P < 0.05; **P < 0.01; ***P < 0.01; ****P < 0.0001.

To further validate the impact of IL-21+IR on the TME, particularly on CD8^+^ T cells, the TMEs of various tumors treated with radiation with or without IL-21 were analyzed based on gating strategies using flow cytometry (Supplemental Figure 6, A–C). The results showed that in the TME of MC38 tumors, the combination of IL-21 and radiation significantly enhanced the infiltration of CD45^+^ and CD8^+^ T cells (Figure 4A). Further examination of the function of tumor-infiltrating CD8^+^ T cells revealed that the expression of cytotoxic markers such as granzyme B (Gzmb), IFN-γ, and TNF-α, as well as the markers for effector CD8^+^ T cells, PD-1, and CD69, were significantly upregulated in the IL-21+IR group (Figure 4A, Supplemental Figure 6, D and E, and Supplemental Figure 7A). In contrast, combination treatment failed to affect the infiltration of exhausted CD8^+^ T cells (Tim3^+^CD8^+^ T cells and PD-1^+^Tim3^+^CD8^+^ T cells) in the TME (Supplemental Figure 7A). Analysis of the TMEs of 2 other tumors that were less immunogenic, CMT167 and MCA205, and 1 “cold” tumor, B16, yielded similar results; the combination therapy promoted the infiltration of CD45^+^ immune cells and CD8^+^ T cells in the TME, particularly the enrichment of effector CD8^+^ T cells characterized by high expression of Gzmb, IFN-γ, and TNF-α (Figure 4, B–D, Supplemental Figure 6, D and E, Supplemental Figure 7B, and Supplemental Figure 8). These findings corroborated the data from the scRNA-Seq analysis. Flow cytometry analysis showed that the combination of IL-21 and radiation led to the downregulation of B cell infiltration across the 4 tumor models and reduced infiltration of tumor-associated macrophages in the TME of MC38 tumors and MCA205 tumors (Supplemental Figure 7, A and B, and Supplemental Figure 8, A and B). In contrast, the remodeling effects of the combination treatment on DCs and myeloid-derived suppressor cells (MDSCs) varied among different tumors. For instance, in MC38 tumors, IL-21+IR significantly reduced the proportion of DCs and increased the proportion of polymorphonuclear MDSCs (Supplemental Figure 7A). In contrast, in CMT167 tumors, the combination treatment increased the proportion of DCs and decreased the proportion of polymorphonuclear MDSCs (Supplemental Figure 7B), while in the MCA205 tumors, the combination treatment primarily promoted the infiltration of monocytic MDSCs and reduced DC infiltration (Supplemental Figure 8A). In B16 tumors, only increased enrichment of monocytic MDSCs was observed in the combination treatment group (Supplemental Figure 8B). These distinctive effects on various cell subsets may be attributed to the heterogeneity of different TMEs and various responses to radiation. To prominently illustrate the impact of IL-21+IR on CD8^+^ T cells in the TME, multicolor immunofluorescence was used to examine MC38 tumors treated with radiation in the presence or absence of IL-21. These findings were consistent with the flow cytometry results, revealing that the combination of IL-21 and radiation significantly increased the infiltration of CD8^+^ T cells and the expression of Gzmb and PD-1, whereas the expression of IL-21 and Tim-3 did not show significant changes (Supplemental Figure 9, A and B). Collectively, these findings demonstrate that the combination of IL-21 and radiation elevates the antitumor function of effector CD8^+^ T cells in the TME.

### IL-21 directly amplifies the activation and function of CD8^+^ T cells.

**Figure 5:**
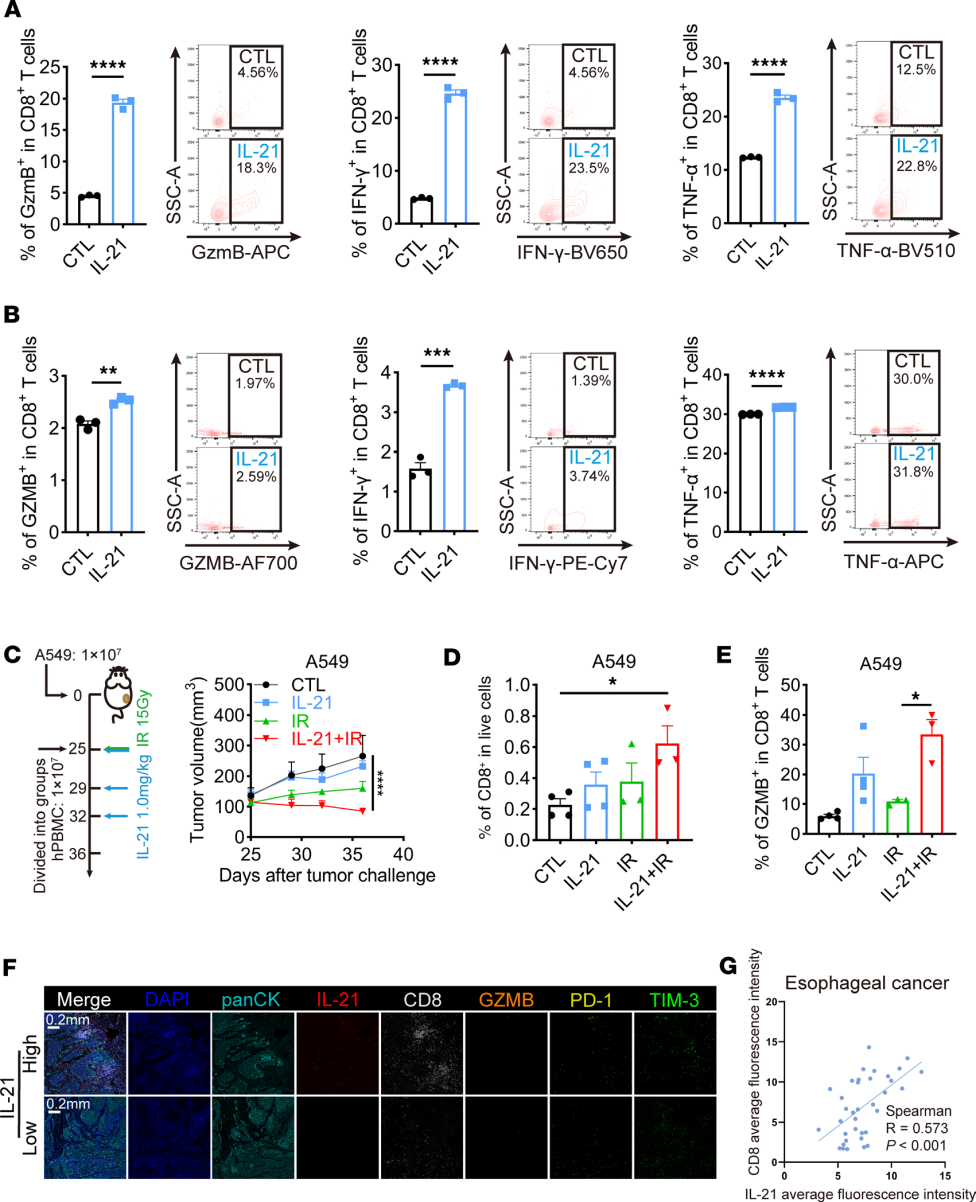
IL-21 directly boosts the activation and cytotoxicity of CD8^+^ T cells. (**A**) Quantitative analysis and representative data of GzmB^+^, IFN-γ^+^, and TNF-α^+^ murine CD8^+^ T cells stimulated with anti-CD3 and anti-CD28 in the presence or absence of IL-21. (**B**) Quantitative analysis and representative data of GZMB^+^, IFN-γ^+^, and TNF-α^+^ human CD8^+^ T cells stimulated with anti-CD3 and anti-CD28 in the presence or absence of IL-21. (**C**) Tumor growth of A549 cells in NSG mice with PBMC engraftment subjected to radiation with or without systematic administration of IL-21. (**D** and **E**) Quantitative analysis of CD8^+^ T cells (**D**) and GZMB^+^CD8^+^ T cells (**E**) from A549 tumors treated with radiation with or without IL-21 by flow cytometry. (**F**) Representative immunofluorescence data of IL-21, CD8, GZMB, PD-1, TIM-3, and panCK expression in radiotherapy-treated esophageal squamous cell carcinoma tissues. Scale bar: 0.2 mm. (**G**) Correlation analysis of the MFI of IL-21 and CD8 of radiotherapy-treated esophageal squamous cell carcinoma tissues. Data shown as mean ± SEM in **A**–**E** (*n* = 3–4 per group) and representative data are shown from 2–3 independent experiments. Statistical analysis was performed using an unpaired 2-tailed Student’s *t* test (**A** and **B**), 2-way ANOVA with Tukey’s multiple-comparison test (**C**), and 1-way ANOVA with Tukey’s multiple-comparison test (**D** and **E**). *P < 0.05; **P < 0.01; ***P < 0.001; ****P < 0.0001.

Previous studies have reported that IL-21 can act directly on CD8^+^ T cells to enhance their function. Therefore, we hypothesized that exogenous administration of IL-21 augments radiation efficacy via direct action on CD8^+^ T cells. To validate this hypothesis, primary CD8^+^ T cells isolated from mouse spleens and human peripheral blood were stimulated in vitro with anti-CD3 and anti-CD28, with or without the addition of IL-21. Flow cytometry analysis revealed that IL-21 significantly enhanced the expression of effector and cytotoxic markers in both mouse and human CD8^+^ T cells, such as Gzmb/GZMB, IFN-γ, TNF-α, and CD69, whereas markers of exhausted CD8^+^ T cells did not show significant changes (Figure 5, A and B, and Supplemental Figure 9C). These results suggest that IL-21 can directly promote the activation of CD8^+^ T cells, consistent with previous reports. To further verify that IL-21 could activate the antitumor immune responses of human CD8^+^ T cells induced by radiation, humanized mouse tumor models were established using the human lung adenocarcinoma cell line A549 and PBMCs from healthy donors, and the tumor growth showed that the combination treatment of IL-21 and radiation induced synergistic antitumor effects (Figure 5C). Moreover, the addition of IL-21 enhanced the infiltration and function of CD8^+^ T cells in the TME (Figure 5, D and E, and Supplemental Figure 9D). Notably, the addition of IL-21 inhibited CD8^+^ T cell exhaustion to a certain degree (Supplemental Figure 9D). Additionally, in A549 tumor–bearing immunodeficient NSG mice, IL-21 failed to show synergic antitumor effects combined with radiation (Supplemental Figure 9E), suggesting that IL-21 enhances radiation-induced antitumor efficacy via immune activation. This was further validated in surgical samples from patients with esophageal squamous cell carcinoma who received neoadjuvant radiotherapy. Multicolor immunofluorescence results showed that the expression of IL-21 in tumor tissues after radiotherapy was significantly positively correlated with the expression levels of CD8 and GZMB and negatively correlated with TIM-3, the exhaustion marker of CD8^+^ T cells (Figure 5, F and G, and Supplemental Figure 9F). In summary, these results indicate that IL-21 can enhance the antitumor effect of radiation by promoting the effector function and inhibiting the exhaustion of CD8^+^ T cells.

## Discussion

Our study proposes the role and mechanism of IL-21 as an immunomodulatory agent in enhancing the antitumor efficacy of radiation, building upon the known research on IL-2 family members in antitumor studies. Our findings indicate that high expression of both IL-21 and IL-21R in the TME is positively correlated with the prognosis of radiotherapy and with the infiltration and function of CD8^+^ T cells. In various murine tumor models, exogenous administration of IL-21 combined with radiation leads to synergistic antitumor effects, which are dependent on the positive regulatory effects of IL-21 on effector CD8^+^ T cells in the TME. Our research not only confirms the potential effects of IL-21 in augmenting the efficacy of radiotherapy but also provides significant experimental evidence for IL-21 as an important biomarker for predicting the effectiveness of radiotherapy.

Radiation therapy remains a cornerstone in the treatment of solid tumors (1); however, advancements in its efficacy have reached a critical juncture, necessitating further exploration. Three primary avenues have emerged to enhance the effectiveness of radiation therapy: refinement of radiation delivery techniques (32), augmentation of radiosensitivity (33), and enhancement of immune cell functionality within the cancer-immunity cycle (34). Targeting immune cells, particularly CD8^+^ T cells, macrophages, and antigen-presenting cells, has shown promise for improving treatment outcomes (11, 35, 36). Although recent investigations into the cytokine milieu, specifically the IL-2 family (IL-2, IL-15, and IL-21), have revealed a burgeoning interest in cytokine-based therapies (19, 37, 38), the synergistic potential of these cytokines combined with radiotherapy remains largely unexplored. Our study conducted a prognostic analysis of various cancer types in the Kaplan-Meier plotter database, TCGA database, and our cohort and found that among the IL-2 family members, only IL-21 and its corresponding receptor IL21-R was positively correlated with radiotherapy prognosis. This can be partially attributed to the immunosuppressive properties of IL-2, IL-4, IL-7, IL-9, and IL-15. For instance, IL-2 facilitates the recruitment of Tregs (17), while persistent IL-15 may lead to NK cell dysfunction (39), and increased expression of IL-4, IL-7, and IL-9 within the TME could foster a protumoral setting (40–42). Furthermore, the analysis of the TME of tumor samples from patients with esophageal squamous cell carcinoma after radiotherapy in our cohort also revealed that the expression of IL-21 is positively correlated with the infiltration of effector CD8^+^ T cells, which is often linked to improved survival in various cancers (43). Our study demonstrates that IL-21 in the TME may serve as a vital prognostic marker for the efficacy of radiotherapy, though the expression of IL-21 in the serum of patients still needs further investigation.

It is well acknowledged that radiation therapy not only kills tumor cells directly exposed to radiation but can also cause the regression of nonirradiated tumors, known as the abscopal effect (1). The abscopal effect induced by radiation therapy alone is uncommon in clinical settings and requires immunotherapy as a facilitator to enhance the radiation-induced abscopal effect (44). Using different subcutaneous and orthotopic tumor models in mice, we determined that exogenous administration of IL-21 combined with radiation could not only lead to more pronounced irradiated tumor regression, but also induce significant abscopal effects. Therefore, our study provides preclinical evidence for the use of a combination of IL-21 and radiation. As demonstrated in a lot of preclinical models and a few clinical cases, hypofractionated radiotherapy (e.g., 8 Gy × 3, 12 Gy × 1, 15 Gy × 1, 20 Gy × 1, 24 Gy × 1) could elicit robust CD8^+^ T cell–dependent antitumor immunity (45–52). Therefore, in our study, we used 15 Gy × 1 for subcutaneous tumor models and 8 Gy × 1 for lung orthotopic tumor models, which mimics the hypofractionated or ablative radiotherapy in clinical practices, to evaluate the synergetic antitumor effects of IL-21 and possible mechanisms. However, conventional radiotherapy, that is 1.8–2.0 Gy per fraction given in multiple fractions with total 50–70 Gy, is most commonly employed in clinical practices. Given that the immunomodulatory consequences of conventional radiotherapy could be different from hypofractionated radiotherapy (6), the combination of IL-21 and conventional radiotherapy needs further experimental exploration and clinical validation. On the other hand, although in vivo experiments found that IL-21-αHSA showed no obvious toxicity in our study, whether IL-21-αHSA could exhibit similar drug safety in humans has yet to be explored in clinical trials.

Similar to other IL-2 family members, IL-21 is a crucial cytokine that modulates the function of diverse immune cells (19). In our study, single-cell transcriptome sequencing and flow cytometry analysis revealed that IL-21 primarily synergizes with radiation to enhance the therapeutic efficacy by augmenting the function of effector CD8^+^ T cells. In vitro experiments suggested that this effect might be mediated through the direct action of IL-21 on CD8^+^ T cells, which is consistent with previous reports. Previous studies have demonstrated that activation of the JAK/STAT pathway (particularly STAT1, STAT3, STAT4) (53, 54), the upregulation of key transcription factors controlling the development and function of CD8^+^ T cells (e.g., T-bet, Eomes) (55, 56), and the metabolically quiescent state of oxidative phosphorylation in CD8^+^ T cells (57) are important mechanisms by which IL-21 promotes the proliferation and function of CD8^+^ T cells, but our scRNA-Seq analysis failed to enrich the aforementioned pathways. However, in the combination treatment (IL-21+IR) group, the phosphatidylinositol signaling system and inositol phosphate metabolism were significantly enriched in effector CD8^+^ T cells. All these finding suggest that the specific mechanisms by which IL-21 enhances the function of effector CD8^+^ T cells in the context of radiation may differ from those in previous reports, possibly because radiation itself could reshape CD8^+^ T cells in the TME through various mechanisms, although further experiments are needed to confirm this. Additionally, previous studies have found that tumor-draining lymph nodes are pivotal sites for the activation of CD8^+^ T cells, especially tumor antigen–specific CD8^+^ T cells, and are also an important source of CD8^+^ T cells infiltrated in the TME (48, 58, 59). Earlier studies have shown that intratumoral administration of IL-21 not only enhances the function of tumor-infiltrating lymphocytes but also boosts the function of various immune cells (CD4^+^ T cells, CD8^+^ T cells, B cells, etc.) in draining lymph nodes (22, 60, 61). These results indicate that under radiation conditions, IL-21 may also enhance the activation and function of CD4^+^ and CD8^+^ T cells in the draining lymph nodes. However, in this study, we did not detect the function of immune cells in the draining lymph nodes after combined treatment, which warrants further exploration in subsequent studies.

In summary, our research indicates that the exogenous administration of IL-21 can enhance radiation efficacy by elevating the function of effector CD8^+^ T cells within the TME. Our study provides significant experimental evidence for the employment of IL-21 as an immunomodulatory agent in combination with radiotherapy. However, further research is required to elucidate the mechanisms by which IL-21 augments CD8^+^ T cell function in the context of radiation. Additionally, the clinical safety of IL-21-αHSA used in our study alone and in combination with radiotherapy needs to be further validated through clinical trials.

## Methods

### Sex as a biological variable.

Human samples were deliberately sourced from male and female individuals. For mouse experiments, to avoid interference from androgen in antitumor effects, only female animals were used in this study.

### Mice.

Female C57BL/6J and BALB/c mice (female, 7 weeks) were purchased from Beijing Vital River Laboratory Animal Technology Co., Ltd. Female NSG mice (age, 7 weeks) were purchased from SPF Biotechnology Co., Ltd. All mice were fed under specific pathogen–free conditions in the animal facilities of Shandong Cancer Hospital and Institute, affiliated with Shandong First Medical University and Shandong Academy of Medical Sciences (temperature: 22°C; humidity: 40%–60%; with free water and fed a 12-hour light/12-hour dark cycle).

### Cell lines and culture condition.

The murine colorectal cancer cell line MC38 and the melanoma cell line B16F10 were provided by Liufu Deng (Shanghai Jiao Tong University, School of Pharmacy, Shanghai, China). The murine fibrosarcoma cell line MCA205 was purchased from the BeNa Culture Collection (BNCC339717). The murine breast cancer cell line 4T1, murine colorectal cancer cell line CT26, and murine melanoma cell line B16 were obtained from the Cell Bank of the Chinese Academy of Sciences (SCSP-5056, SCSP-523, and SCSP-5096). The murine lung carcinoma cell line CMT167 was purchased from Otwo Biotech Inc. (HTX3130). Luciferase-expressing CMT167 (CMT167-luci) cells were established using lentiviruses purchased from GenePharma (2022-38569). The MC38 cells, CMT167 cells, 4T1 cells, and CT26 cells were cultured in DMEM (Thermo Fisher Scientific, C11995500BT) supplemented with 10% FBS (ExCellBio, FSP500) and 100 U/mL penicillin/streptomycin (Solarbio, P1400) at 37°C with 5% CO_2_. B16, B16F10, and MCA205 cells were cultured in RPMI-1640 (Thermo Fisher Scientific, C11875500BT) supplemented with the same ingredients.

### CD8^+^ T cell isolation and culture.

To determine the effects of IL-21 on the function of mouse and human CD8^+^ T cells in vitro, mouse CD8^+^ T cells were obtained from the spleens of C57BL/6J mice (7 weeks old) through negative selection using the EasySep Mouse CD8^+^ T Cell Isolation Kit (STEMCELL Technologies, 19853), and human CD8^+^ T cells were obtained from the peripheral blood of healthy donors using the EasySep Human CD8^+^ T Cell Isolation Kit (STEMCELL Technologies, 17953) according to the manufacturer’s standard protocols. Subsequently, anti-mouse/human CD3 (2 μg/mL, BioGems, Inc., 05111-20 and 05112-25) and anti–mouse/human CD28 (5 μg/mL, BioGems, Inc., 10311-20 and 10312-25) were used to stimulate CD8^+^ T cells in the presence or absence of IL-21 (Shanghai Junshi Biosciences Co., Ltd., JS014, 5 μg/mL), followed by flow cytometry analysis after 48 hours.

### Mouse models and treatment.

Unilateral subcutaneous tumor models were established using MC38, MCA205, B16, B16F10, CMT167, 4T1, and CT26 cells (1 × 10^6^ cells) as previously reported. Briefly, the indicated cells resuspended in 100 μL PBS were subcutaneously injected into the right flank of C57BL/6J or BALB/c mice. For the bilateral subcutaneous tumor models, 1 × 10^6^ MC38 cells or B16 cells were subcutaneously inoculated in the right flank as the first tumor followed by radiation, and 5 × 10^5^ MC38 cells or B16 cells were subcutaneously inoculated in the left flank on the same day as the second tumor. When the unilateral tumor or first tumor volume reached approximately 100 mm^3^, the mice were randomly grouped and intraperitoneally administered either IL-21 (Shanghai Junshi Biosciences Co., Ltd., JS014) or PBS (Solarbio, P1020) of equal volume twice a week, and the unilateral tumors or first tumors were irradiated with 1 fraction of 15 Gy using the RS2000-225 Biological Irradiator (Rad Source Technologies, Inc.) the next day. Tumor volume was monitored at least twice a week with calipers and calculated using *V* = (length × width^2^)/2. The mice were euthanized at the indicated time or when the tumor volume reached 1,500 mm^3^.

An orthotopic lung cancer model was established using CMT167-luci cells from C57BL/6J mice. First, 5 × 10^4^ CMT167-luci tumor cells resuspended in PBS were mixed with Matrigel (Corning, 356234) at a ratio of 1:1 (5 μL/mouse) and injected directly into the left mouse lung on day 0. The tumor-bearing mice were then randomly grouped and intraperitoneally administered IL-21 (twice per week) from day 6 (total of 9 doses), followed by irradiation on lung tumors with 1 fraction of 8 Gy using the Small Animal Radiation Research Platform (Xstrahl, SN2019077) on day 7. Tumor size and luciferase signal were monitored using IRIS PET/CT (Inviscan SAS) and IVIS Spectrum CT (PerkinElmer Inc.), respectively.

For in vivo depletion of CD8^+^ T cells, tumor-bearing mice were intraperitoneally administered an anti–mouse CD8α antibody (200 μg/mouse, BioXcell, BE0061) 2 days before and twice weekly thereafter. For triple therapy, tumor-bearing mice were intraperitoneally administered anti–PD-1 antibody (200 μg/mouse, BioXcell, BE0146) or solvent of equal volume with or without simultaneous administration of IL-21 twice every week, followed by irradiation 1 day after the first administration of IL-21 or anti–PD-1. Tumor volume was monitored and calculated as above. The mice were euthanized at the indicated time or when the tumor volume reached 1,500 mm^3^.

Humanized mice were established as previously reported (62, 63). Briefly, A549 cells (1 × 10^7^) were subcutaneously inoculated into the right flank of the NSG mice on day 0. When the mean volume of the tumors reached approximately 100 mm^3^ on day 25 after tumor challenge, 1 × 10^7^ PBMCs were obtained using human lymphocyte separation fluid (Lymphoprep Density Gradient Medium, STEMCELL Technologies Inc., 07851) from healthy donors according to the manufacturer’s standard protocols via density gradient centrifugation and introduced into mice via tail vein injection. Subsequently, the mice were randomly grouped and administered IL-21 (1.0 mg/kg, twice a week, a total of 3 doses administered on days 25, 29, and 32) with or without irradiation of 1 fraction of 15 Gy after PBMC transplantation on day 25. Tumor volume was monitored and calculated as above; mice were euthanized 37 days after tumor inoculation and subsequently subjected to flow cytometry assay of the TME. To evaluate the antitumor effects of the combination of IL-21 and radiation in immunodeficient NSG mice without PBMC engraftment, A549 cells (1 × 10^7^) were subcutaneously inoculated into the right flank of the NSG mice. When the tumor volume reached 100 mm^3^, the mice were randomly grouped and intraperitoneally administered either IL-21 (Shanghai Junshi Biosciences Co., Ltd., JS014) or PBS (Solarbio, P1020) of equal volume twice a week with or without radiation treatment of 1 fraction of 15 Gy as described above. Tumor volume was monitored and calculated as above. The mice were euthanized at the indicated time or when the tumor volume reached 1,500 mm^3^.

### Flow cytometry.

Subcutaneous tumors were collected 8–10 days after tumor irradiation, minced with ophthalmic forceps, and subsequently digested with 1 mg/mL collagenase type 4 (Worthington Biochemical Corp., LS004186) and 0.2 mg/mL DNase I (Sigma-Aldrich, DN25) at 37°C for 30 minutes. Digestion was terminated using RPMI-1640 with 2% FBS, and the solution was filtered using a 70 μm cell strainer (Biosharp Life Sciences, BS-70-CS) to obtain single-cell suspensions, followed by cell staining and flow cytometry. For surface staining, the single-cell suspensions from mice were incubated with anti–mouse CD16/CD32 antibody (1:50, BD Biosciences, 553141) for 15 minutes at 4°C to block nonspecific antibody binding, washed with FACS buffer (2% FBS in PBS), and subsequently stained with an antibody cocktail against Fixable Viability Stain 780 (1:1,000, BD Biosciences, 565388), CD45.2 (1:200, BioLegend, 103149; 1:200, BD Biosciences, 552848), CD3 (1:200, BioLegend, 1100204/100233; 1:200, BD Biosciences, 552774), CD8 (1:200, BioLegend, 100730), CD4 (1:200, BioLegend, 100546/100451), CD11b (1:200, BioLegend, 117310), CD11c (1:200, BioLegend, 117349), CD19 (1:200, BioLegend, 115506), MHC-II (1:200, BioLegend, 107626), Ly6C (1:200, BioLegend, 128033), Ly6G (1:200, BioLegend, 127639), F4/80 (1:200, BioLegend, 123132), CD69 (1:200, BioLegend, 104505), CD279 (1:200, BioLegend, 135231), and CD366 (1:200, BioLegend, 134012). For intracellular staining to evaluate the function of T cells in the TME, single-cell suspensions were treated with Cell Activation Cocktail (with Brefeldin A) (1:200, BioLegend, 423304) for 4 hours before cell staining and stained with an antibody cocktail against CD45.2 (1:200, BioLegend, 103149) and CD8 (1:200, BioLegend, 100730). The samples were fixed in fixation/permeabilization buffer (Thermo Fisher Scientific, 00-5523-00) at 4°C for 1 hour, washed with 1× permeabilization/wash buffer, and incubated with the corresponding antibody cocktails against GZMB (1:200, BioLegend, 372204/396410) and IFN-γ (1:200, BioLegend, 505831) in permeabilization buffer in the dark at 4°C for 40–60 minutes.

To determine the effects of IL-21 on human CD8^+^ T cells in the TME after radiotherapy, A549 tumors from humanized mice were collected 37 days after the tumor challenge. Single-cell suspensions were prepared as described above and then subjected to surface staining against Fixable Viability Stain 780, CD45 (1:200, BioLegend, 304047), CD3 (1:200, BioLegend, 317323), CD4 (1:200, BioLegend, 317435), CD8 (1:200, BioLegend, 344747), PD-1 (1:200, BioLegend, 367428), and TIM-3 (1:200, BioLegend, 345016), followed by intracellular staining against GZMB (1:200, BioLegend, 372221), TNF-α (1:200, BioLegend, 502913), and IFN-γ (1:200, BioLegend, 502527).

To analyze the effects of IL-21 on mouse CD8^+^ T cells in vitro, the stimulated cells were subjected to surface staining against Fixable Viability Stain 780, CD45.2 (1:200, BioLegend, 103149), CD8 (1:200, BioLegend, 100730), and CD69 (1:200, BioLegend, 104505), followed by intracellular staining against GZMB (1:200, BioLegend, 372204), IFN-γ (1:200, BioLegend, 505831), TNF-α (1:200, BioLegend, 506339), PD-1 (1:200, BioLegend, 135231), and Tim-3 (1:200, BioLegend, 134012). Similarly, the stimulated human CD8^+^ T cells were stained with Fixable Viability Stain 780, CD8 (1:200, BioLegend, 344747), GZMB (1:200, BioLegend, 372221), TNF-α (1:200, BioLegend, 502913), IFN-γ (1:200, BioLegend, 502527), PD-1 (1:200, BioLegend, 367428), and TIM-3 (1:200, BioLegend, 345016).

Flow cytometry was performed on an LSR Fortessa system (BD Biosciences), and the output was analyzed using FlowJo software (version 10.4).

### scRNA-Seq.

Single-cell suspensions (prepared as mentioned above) were obtained 9 days after MC38 tumor irradiation. Tumor-infiltrating lymphocytes were isolated via density gradient centrifugation using Ficoll (GE Healthcare, 17-5442-02). The obtained tumor-infiltrating lymphocytes were stained with an antibody cocktail against Fixable Viability Stain 510 (1:1,000, BD Biosciences, 564406) and CD45.2 (1:200, BioLegend, 103116). Live CD45^+^ immune cells were sorted using a FACSAria III (BD Biosciences), followed by single-cell transcriptome sequencing using the droplet-based 10× Genomics platform (CapitalBio). Stringent data quality-control was conducted during downstream analysis. The initial dataset contained 64,010 cells, which were filtered using the following parameters to exclude outliers: maximum percentage mito (Percent_mito) = 20%, maximum number of unique molecular identifiers = 60,000, minimum number of genes = 300, and maximum number of genes = 7,500 (64). After discarding poor-quality cells, 27,818 cells were retained for downstream analyses. T-distributed stochastic neighbor embedding (t-SNE) plots and uniform manifold approximation and projection (UMAP) plots were drawn based on the single-cell analysis platform provided by CapitalBio (follow-up visual analysis was also based on the platform). The cluster/subcluster was preliminarily defined through singleR (ImmGenData), and each cell type was determined based on the multiple cell type–specific marker genes identified in the previous literature (64–66) as follows: *Cd3e*, *Cd3d*, and *Cd3g* for T cells; *Cd8a* for CD8^+^ T cells; *Cd4* for CD4^+^ T cells; *Cd4* and *Foxp3* for Treg cells; *Cd19*, *Ms4a1*, and *Cd79a* for B cells; *Ly6c2*, *Ccr2*, and *S100a4* for monocytes; *Adgre1*, *Cd68*, and *Arg1* for macrophages; *S100a8*, *S100a9*, and *Retnlg* for neutrophils; *Ccl22*, *Cd209a*, and *Batf3* for DCs; and *Gzma*, *Klrb1c*, and *Ncr1* for NK cells. Subsequently, Nightingale rose charts and heatmaps were used to illustrate the proportions of different immune cell types. For deep analysis of CD8^+^ T cells, 4 subclusters were identified after removal of low-quality data: naive CD8^+^ T cells (*Ccr7*, *Lef1*, and *Tcf7*), effector and memory CD8^+^ T cells (*Gzma*, *Ccl5*, *Cxcr3*, and *Gzmk*), exhausted CD8^+^ T cells (*Pdcd1*, *Tnfrsf9*, *Ifng*, and *Havcr2*), and proliferating CD8^+^ T cells (*Mki67* and *Top2a*). Scatter plots were used to display the expression levels and distribution of marker genes in each subset of CD8^+^ T cells. To infer the differentiation trajectories of CD8^+^ T cells, a pseudo-time analysis of individual cells was conducted using Monocle (https://bioconductor.org/packages/release/bioc/html/monocle.html) with the DDRTree algorithm based on characteristic genes. To analyze the function of CD45^+^ immune cells and CD8^+^ T cells from different treatment groups, the analysis was conducted using the GSEA function in clusterProfiler (https://bioconductor.org/packages/release/bioc/html/clusterProfiler.html, 4.4.4) with default parameters (*P*-value cutoff = 0.05) with data from the GO and the KEGG databases. Next, for subgroups of CD8^+^ T cells, the pairwise.wilcox.test() function was used to analyze cytotoxic marker genes and exhaustion marker genes between the IR and IL-21+IR groups.

### Multiplex immunofluorescence assay and analysis.

To further verify the relationship between the expression of IL-21/IL-21R and the TME, multicolor fluorescent immunohistochemistry was performed on lung adenocarcinoma tumor microarrays purchased from ShanDong PhenoScience Biotechnology Ltd., according to standard protocols (Akoya Biosciences, NEL871001KT); the relevant clinical information is listed in Supplemental Table 1. The following markers were used: panCK (1:200, ZSGB-BIO, ZM-0069) labeled with Akoya Opal fluorophores 480, IL-21R (1:50, Proteintech, 10533-1-AP) labeled with Akoya Opal fluorophores 570, CD3 (1:200, Abcam, ab16669) labeled with Akoya Opal fluorophores 690, CD45 (1:300, Proteintech, 20103-1-AP) labeled with Akoya Opal fluorophores 620, IL-21 (1:50, ABclonal Biotechnology Co., Ltd., A7235) labeled with Akoya Opal fluorophores 520, and CD8 (1:300, Abcam, ab199016) labeled with Akoya Opal fluorophores 780, and the nuclei were labeled with DAPI (1:100, Akoya Biosciences). CD3^+^ cells, CD8^+^ cells, CD45^+^ cells, IL-21^+^ cells, IL-21R^+^ cells, and tumor areas were photographed and analyzed using the Vectra Polaris system (Akoya Biosciences).

To further verify the relationship between IL-21 expression and the TME after radiotherapy, multicolor fluorescent immunohistochemistry was conducted on specimens from patients with esophageal squamous cell carcinoma who had undergone neoadjuvant radiotherapy provided by Shandong Cancer Hospital. The relevant clinical information is listed in Supplemental Table 2. The following markers were used: panCK (1:200, ZSGB-BIO, ZM-0069) labeled with Akoya Opal fluorophores 480, PD-1 (1:200, ZSGB-BIO, ZM-0381) labeled with Akoya Opal fluorophores 570, GZMB (1:200, Abcam, ab255598) labeled with Akoya Opal fluorophores 690, TIM-3 (1:1000, Abcam, Ab241332) labeled with Akoya Opal fluorophores 520, IL-21 (1:50, ABclonal Biotechnology Co., Ltd., A7235) labeled with Akoya Opal fluorophores 620, CD8 (1:300, Abcam, ab199016) labeled with Akoya Opal fluorophores 780, and the nuclei were labeled with DAPI (1:100, Akoya Biosciences). CD8^+^ cells, PD-1^+^ cells, TIM-3^+^ cells, IL-21^+^ cells, GZMB^+^ cells, and tumor areas were photographed and analyzed using the Vectra Polaris system (Akoya Biosciences).

To determine the function of IL-21 in the TME after radiotherapy, multicolor fluorescent immunohistochemistry was performed on mouse tumor tissues obtained 9 days after radiation, fixed in formalin, and embedded in paraffin. The following markers were used: GZMB (1:200, Abcam, ab255598) labeled with Akoya Opal fluorophores 480, PD-1 (1:200, Abcam, ab214421) labeled with Akoya Opal fluorophores 570, IL-21 (1:50, ABclonal Biotechnology Co., Ltd., A7235) labeled with Akoya Opal fluorophores 520, IFN-γ (1:1000, ABclonal Biotechnology Co., Ltd., A12450) labeled with Akoya Opal fluorophores 690, TIM-3 (1:1,000, Abcam, ab241332) labeled with Akoya Opal fluorophores 620, CD8 (1:800, Abcam, ab217344) labeled with Akoya Opal fluorophores 780, and the nuclei were labeled with DAPI (1:100, Akoya Biosciences). CD8^+^ cells, PD-1^+^ cells, TIM-3^+^ cells, IL-21^+^ cells, GZMB^+^ cells, and IFN-γ^+^ cells were photographed and analyzed using the Vectra Polaris system (Akoya Biosciences).

### H&E staining.

To explore the safety of systematic administration of IL-21, the vital organs (brain, heart, lung, thymus, liver, kidney, spleen, and lymph nodes) of mice systematically administered IL-21 and subjected to H&E staining. Briefly, the sections were deparaffinized in xylene, followed by hydration by immersion in 100%, 95%, 80%, and 70% ethanol. Subsequently, staining was conducted using hematoxylin staining solution (Beyotime, C0107) and eosin staining solution (Beyotime, C0109). Finally, the sections were subjected to gradient dehydration and mounting and observed under a microscope.

### Statistics.

The data are presented as mean ± SEM and were compared using a 2-tailed Student’s *t* test for 2 groups and 1-way ANOVA followed by multiple-comparison test for more than 2 groups. Tumor curve data were compared using 2-way ANOVA. Survival analysis was performed using the Kaplan-Meier method with a log-rank test. Statistical significance was set at *P* less than 0.05.

### Study approval.

Studies using human samples were approved by the ethics committee of Shandong Cancer Hospital and Institute (no. 202511043) and conform to the guidelines of the 2000 Helsinki Declaration. All animal studies were performed according to the protocol approved by the IACUC of Shandong Cancer Hospital and Institute (no. SDTHEC2024006125).

### Data availability.

All data necessary for the evaluation of the conclusions in the article are presented in the article and/or in the supplemental materials and methods. The survival data of the IL-2 family analyzed in this study can be accessed on the Kaplan-Meier plotter (https://kmplot.com/analysis/) and TCGA database (https://www.cancer.gov/ccg/research/genome-sequencing/tcga). The scRNA-Seq data of MC38 tumors in study are available in the National Genomics Data Center under accession PRJCA052467 (https://ngdc.cncb.ac.cn/bioproject/). Values for all data points in graphs are reported in the Supporting Data Values file.

## Author contributions

DWC, MW, and JMY designed the study. XYL, XQX, MXC, QXT, and JW performed the in vivo animal experiments. XYL, BCW, and RFL conducted the in vitro cell experiments. BCW, SSM, QW, and YHH helped with the flow cytometry experiments. XYL, BCW, and MXC analyzed the scRNA-Seq data. BCW, XZS, XR, LW, and ZYL helped with the data analysis of flow cytometry. XYL and MW wrote the manuscript with input from all authors. DWC, MW, and JMY supervised the study and edited the manuscript. All authors have read and approved the article.

## Funding support

- National Natural Science Foundation of China (82172676 and 82373217 to DWC and 82030082 to JMY).
- Natural Science Foundation of Shandong Province (ZR2021YQ52 and ZR2024JQ032 to DWC, ZR2023ZD26 to JMY, ZR2024MH042 to MW, and ZR2020MH235 to LW).
- Collaborative Academic Innovation Project of Shandong Cancer Hospital (GF001 to DWC).
- Noncommunicable Chronic Diseases-National Science and Technology Major Project (2024ZD0519900 and 2024ZD0519902 to JMY and DWC).
- Beijing Bethune Charitable Foundation (to DWC).
- Key Research and Development Program of Shandong Major Science & Technology Innovation Project (2021SFGC05012021 to JMY).

## Supplementary Material

Supplemental data

Supporting data values

## Version 1 01/08/2026

In-Press Preview

## Version 2 02/23/2026

Electronic publication
