# Supplementary material for: IL-21 enhances the cytotoxicity of intratumoral CD8+ T cells, improving radiation efficacy
Source: JCI Insight. 2026 Jan 8;11(4):e190531. doi: 10.1172/jci.insight.190531 (PMC12956009; doi:10.1172/jci.insight.190531)
Supplement: Supplemental data [file jciinsight-11-190531-s188.pdf]

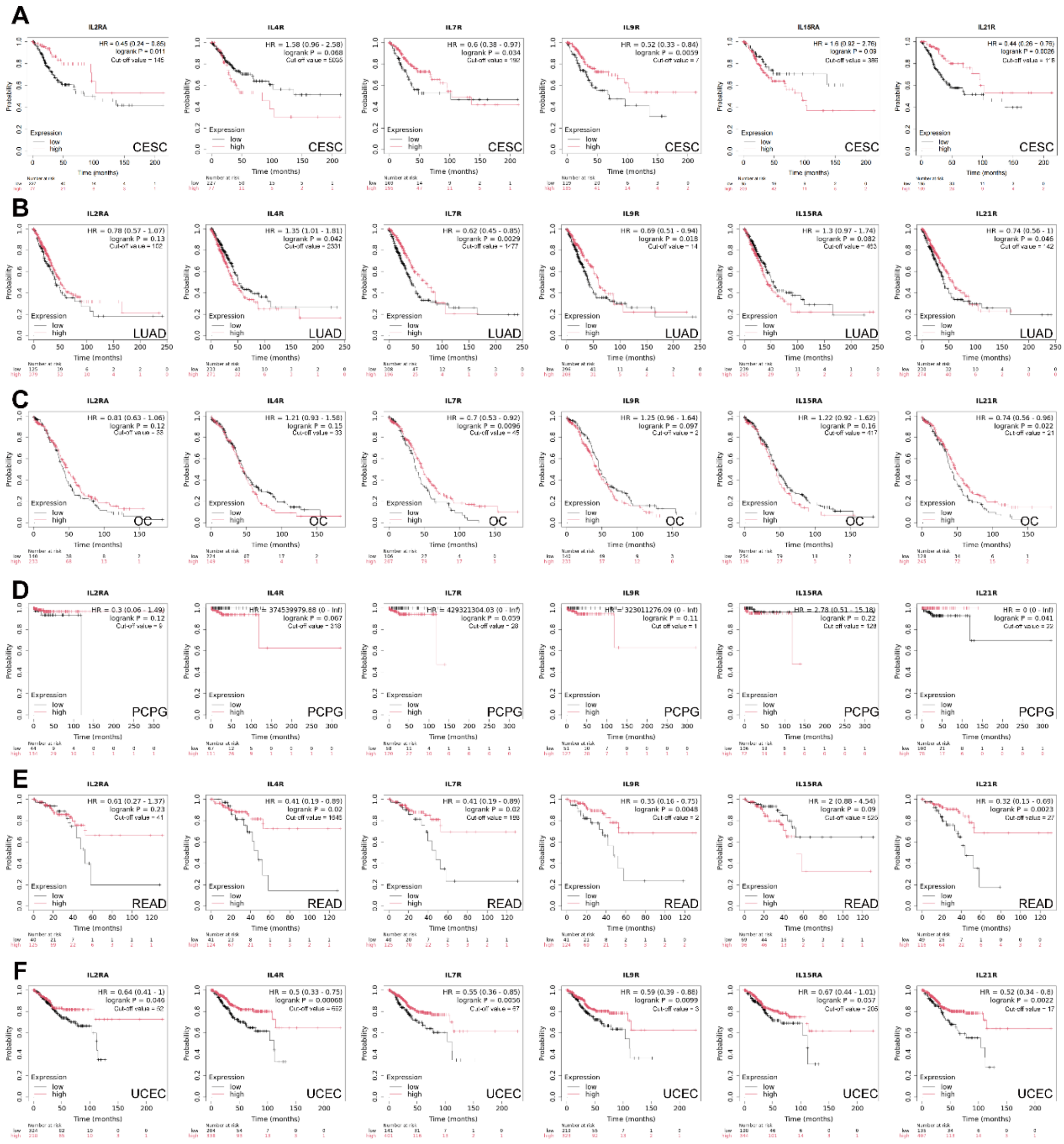

Figure S1. The expression of IL21 in TME is correlated with favorable prognosis.

Kaplan–Meier analysis of the overall survival (OS) of CESC (A), LUAD (B), OC (C), PCPG (D), READ (E), and UCEC (F) patients with high or low expression *IL2RA*, *IL4R*, *IL7R*, *IL9R*, *IL15RA* and *IL21R* from KM-plotter database.

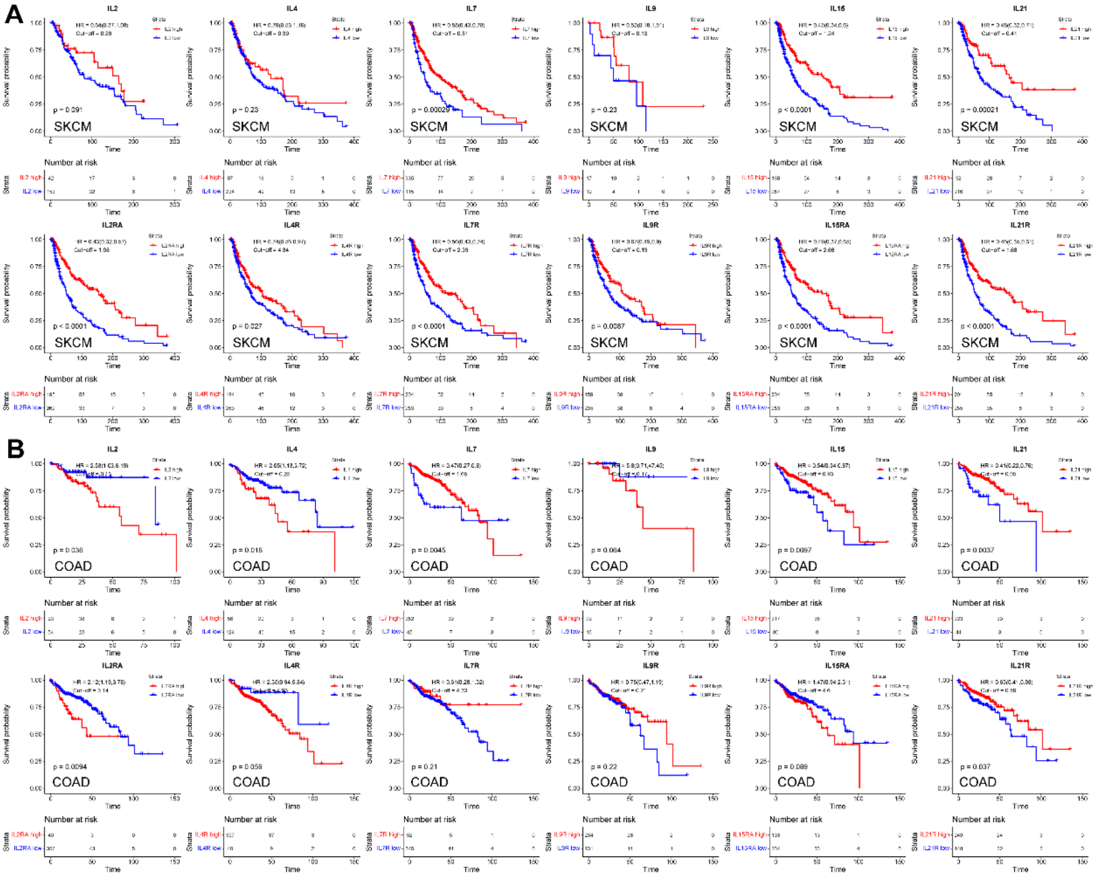

Figure S2. The expression of IL21 in TME is correlated with favorable radiotherapy prognosis. (A) Kaplan–Meier analysis of OS of radiotherapy-treated SKCM patients with high or low expression of *IL2*, *IL4*, *IL7*, *IL9*, *IL15*, *IL21* and corresponding receptors (*IL2RA*, *IL4R*, *IL7R*, *IL9R*, *IL15RA* and *IL21R*) from TCGA database. (B) Kaplan–Meier analysis of OS of radiotherapy-treated COAD patients with high or low expression of *IL2*, *IL4*, *IL7*, *IL9*, *IL15*, *IL21* and corresponding receptors (*IL2RA*, *IL4R*, *IL7R*, *IL9R*, *IL15RA* and *IL21R*) from TCGA database.

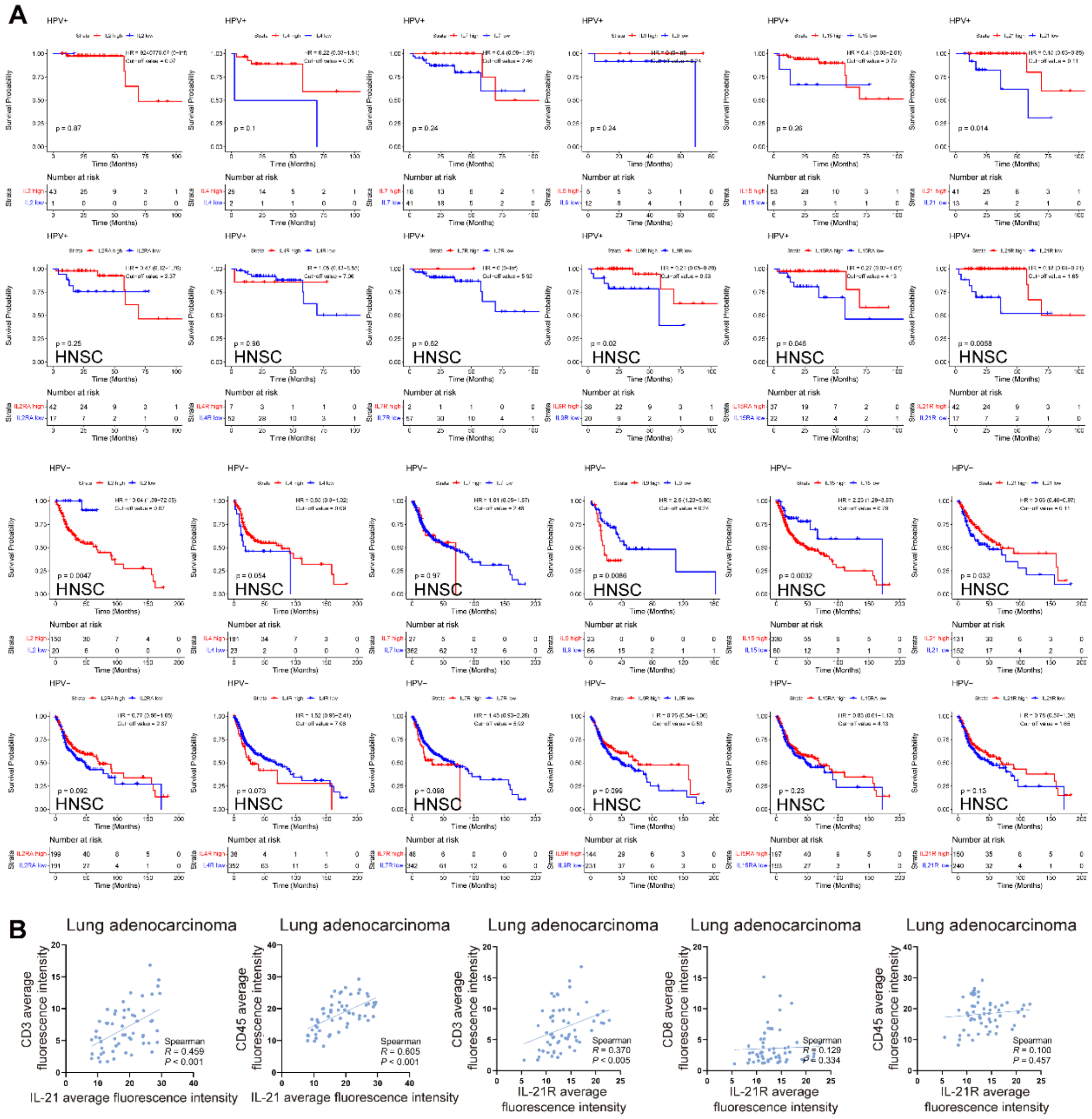

Figure S3. The expression of IL21 in TME is correlated with favorable radiotherapy prognosis of HNSC patients and increased CD8^{+} T cell infiltration. (A) Kaplan–Meier analysis of OS of radiotherapy-treated HPV^{+} and HPV^{-} HNSC patients with high or low expression of *IL2*, *IL4*, *IL7*, *IL9*, *IL15*, *IL21* and corresponding receptors (*IL2RA*, *IL4R*, *IL7R*, *IL9R*, *IL15RA* and *IL21R*) from TCGA database. (B) Correlation analysis of expression of IL-21/IL-21R and CD3/CD8/CD45 in lung adenocarcinoma samples.

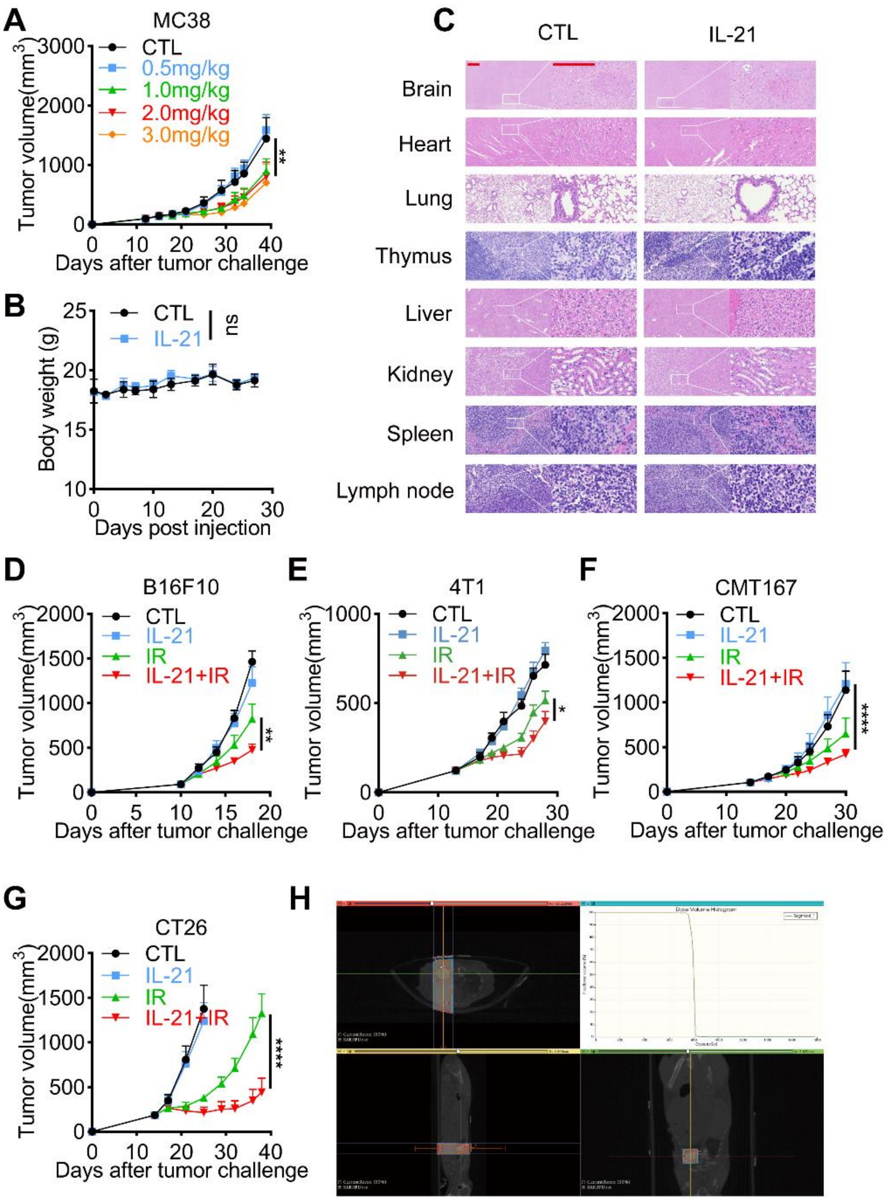

Figure S4. Exogenous IL-21 administration synergistically enhances radiation efficacy with demonstrated safety. (A) Tumor growth of MC38 tumors in C57BL/6J mice subjected to intraperitoneal administration of IL-21 (0.5 mg/kg, 1.0 mg/kg, 2.0 mg/kg, 3.0 mg/kg) (n =7 per group). (B) Body weight of C57BL/6J mice with intraperitoneal administration of PBS or IL-21 (1.0 mg/kg) (n =3 per group). (C) Representative data of HE staining of brain, heart, lung, thymus, liver, kidney, spleen and lymph node of C57BL/6J mice treated with or without IL-21 (1.0 mg/kg). (D-G)

Tumor growth of B16F10, 4T1, CMT167 and CT26 tumors in C57BL/6J and Balb/c mice treated with radiation with or without IL-21 (n =6 per group). (H) Precise radiation target area and dose volume histogram for lung orthotopic tumors. Data are shown as mean ± SEM. Statistical analysis was performed using two-way ANOVA followed by Dunneet's multiple comparison test (A), two-way ANOVA with Sidak's multiple comparison test (B, G), and two-way ANOVA with Tukey multiple comparison test (D-F). *$p<0.05$, **$p<0.01$, ****$p<0.0001$.

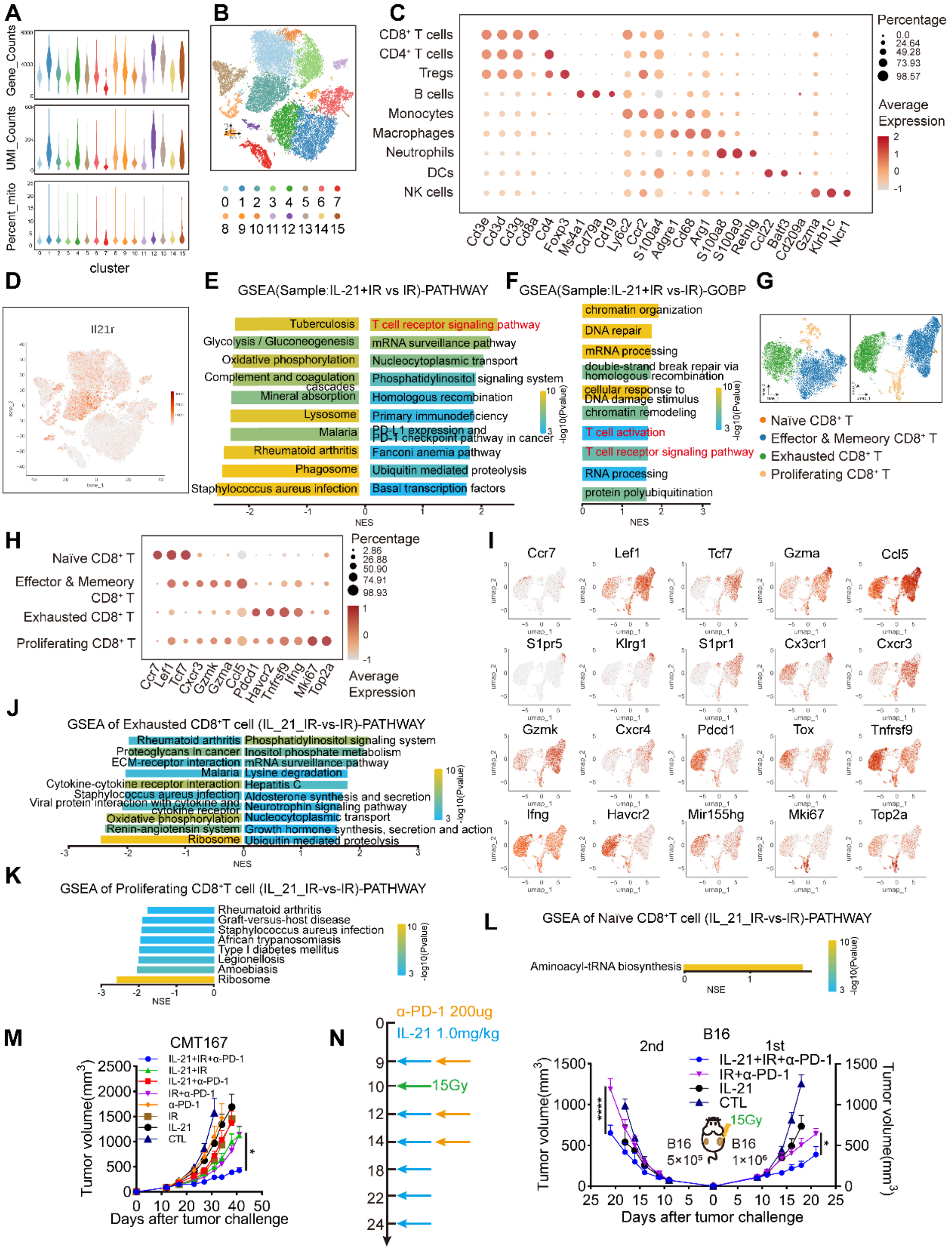

Figure S5. IL-21 combined with radiation reshapes the TME and increases the infiltration of CD8⁺ T cells. (A) Violin plots demonstrating the data quality control of scRNA-seq. Maximum percentage mito=20%, maximum number of UMIs=60,000, minimum number of nGene=300, and maximum number of nGene=7,500. (B) UMAP

plot showing sorted $CD45^+$ immune cells. (C) Bubble Chart illustrating the expression levels of the marker genes in each subset of $CD45^+$ immune cell. (D) t-SNE plot showing the expression levels of *Il21r* in different subtypes of $CD45^+$ immune cells. (E-F) GSEA analysis of upregulated and downregulated genes for $CD45^+$ immune cell in IL-21+IR group *vs* IR group. (G) t-SNE and UMAP plots showing sorted $CD8^+$ T cells. (H) Bubble Chart showing the expression levels of the marker genes in each subset of $CD8^+$ T cells. (I) UMAP plots showing the expression levels of the marker genes in different subsets of $CD8^+$ T cells. (J-L) GSEA analysis of upregulated and downregulated genes for Exhausted (J), Proliferating (K) and Naïve (L) $CD8^+$ T cells in IL-21+IR group *vs* IR group. (M) Tumor growth of CMT167 tumors treated with radiation and/or IL-21 with or without anti-PD-1. (N) Tumor growth of B16 tumors treated with radiation combined with anti-PD-1 with or without IL-21. Panel M and N (n = 5-7 per group) are demonstrated as mean ± SEM. Statistical analysis was carried out using two-way ANOVA with Tukey's multiple comparisons test (M) and two-way ANOVA with Sidak's multiple comparisons test (N). *$p<0.05$, ****$p<0.0001$.

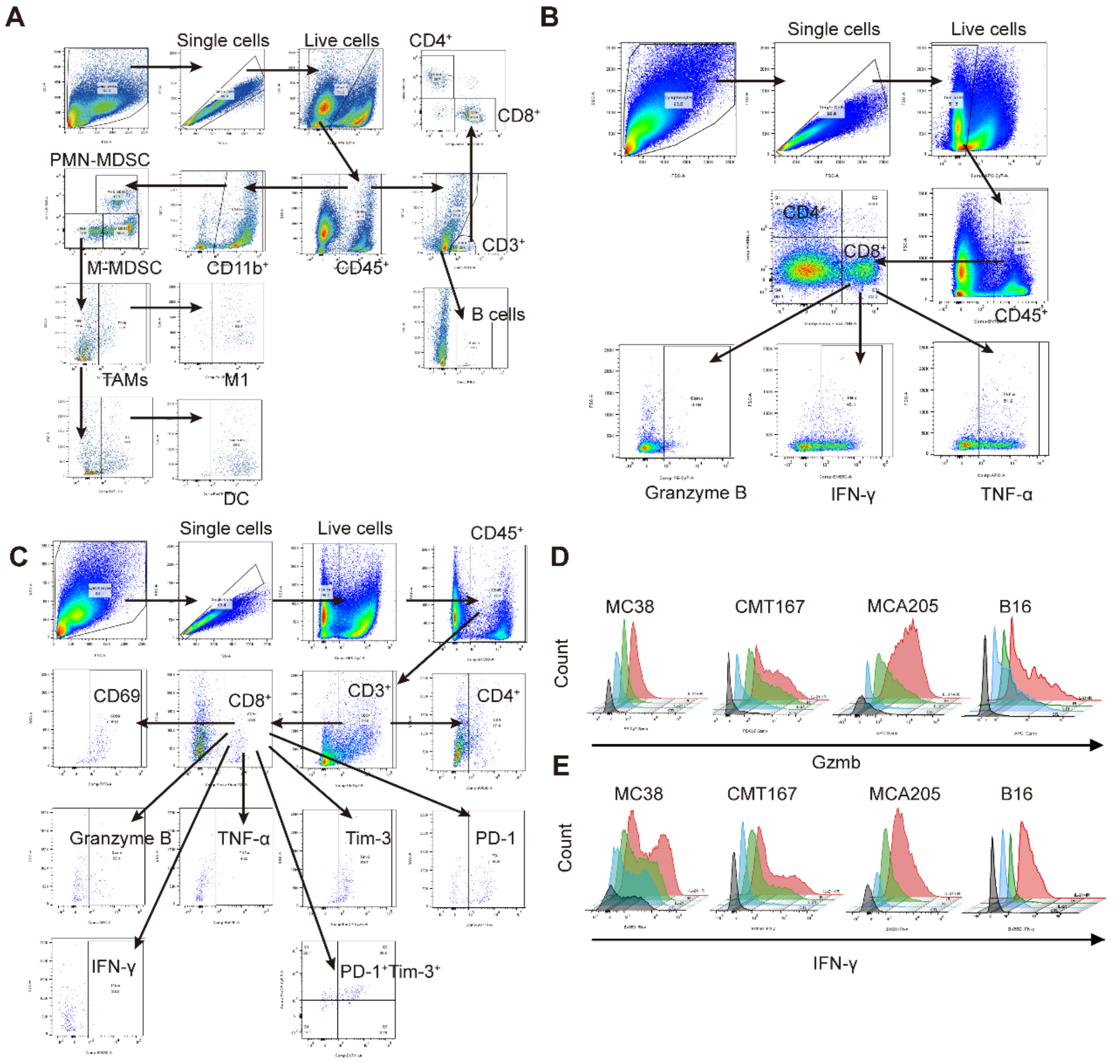

Figure S6. Gating strategies of flow cytometry analysis and flow cytometry histograms of Gzmb and IFN-γ expression in CD8$^+$ T cells. (A) Gating strategy of flow cytometry analysis for CD45$^+$ immune cells in TME. (B) Gating strategy of flow cytometry analysis for CD8$^+$ T cells in TME. (C) Gating strategy of flow cytometry analysis for CD8$^+$ T cells in TME. (D) Expression of Gzmb$^+$ and IFN-γ$^+$ of CD8$^+$ T cells from MC38, CMT167, MCA205 and B16 tumors.

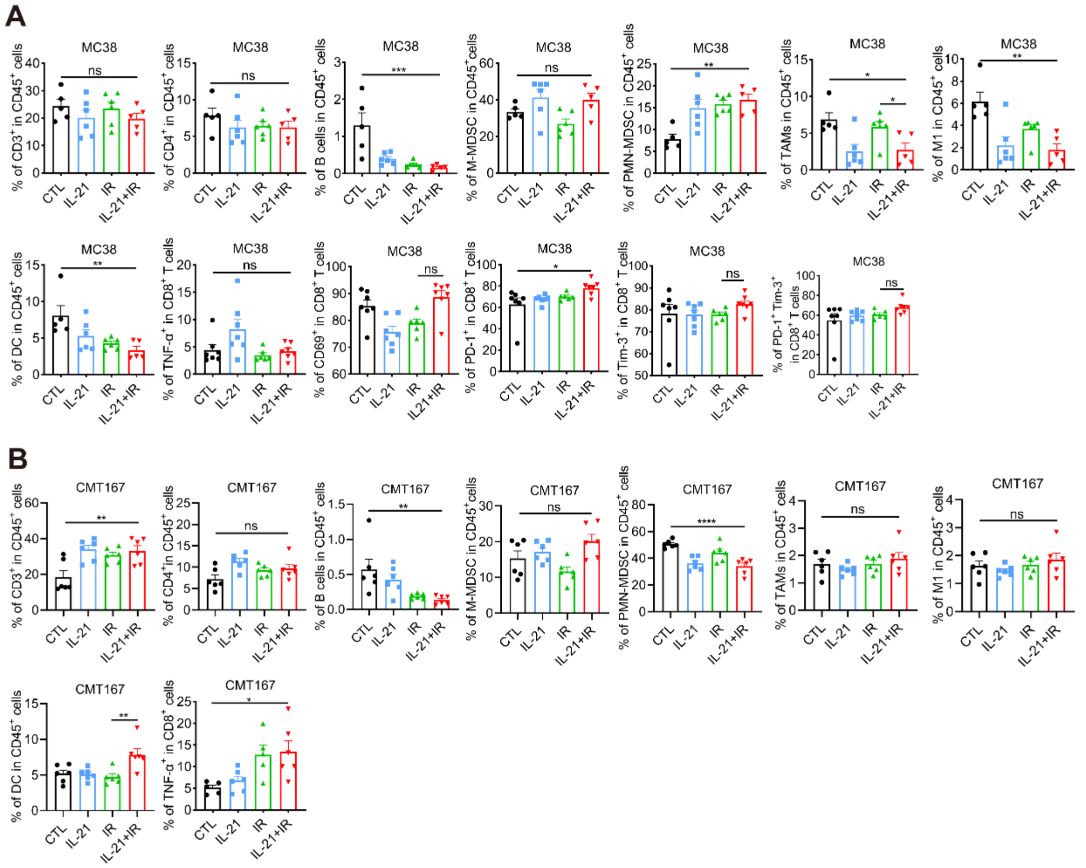

Figure S7. Flow cytometric analysis of different subsets of immune cells in TME of MC38 and CMT167 tumors. (A) Quantitative analysis of $CD3^+$ T cells, $CD4^+$ T cells, B cells, M-MDSCs, PMN-MDSCs, TAMs, M1-TAMs, DCs, TNF-α^+ $CD8^+$ T cells, $CD69^+$ $CD8^+$ T cells, PD-1^+ $CD8^+$ T cells, Tim-3^+ $CD8^+$ T cells and PD-1^+ Tim-3^+ $CD8^+$ T cells from MC38 tumors subjected to radiation with or without IL-21. (B) Quantitative analysis of $CD3^+$ T cells, $CD4^+$ T cells, B cells, M-MDSCs, PMN-MDSCs, TAMs, M1-TAMs, DCs and TNF-α^+ $CD8^+$ T cells from CMT167 tumors subjected to radiation with or without IL-21. Data are shown as mean ± SEM (n = 5-7 per group). Statistical analysis was performed using one-way ANOVA followed by Tukey's multiple comparison tests (A-B). ns, $p>0.05$, *$p<0.05$, **$p<0.01$, ***$p<0.001$.

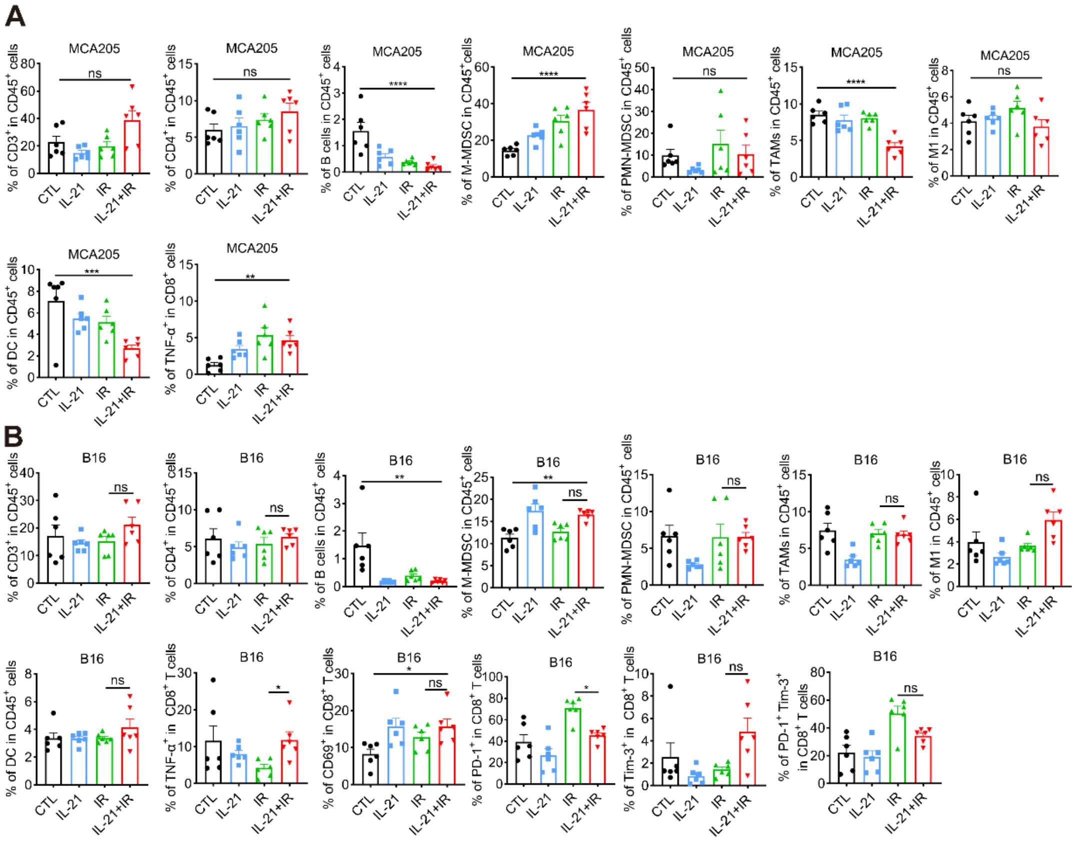

Figure S8. Flow cytometric analysis of different subsets of immune cells in TME of MCA205 and B16 tumors. (A) Quantitative analysis of $CD3^+$ T cells, $CD4^+$ T cells, B cells, M-MDSCs, PMN-MDSCs, TAMs, M1-TAMs, DCs and TNF-α^+ $CD8^+$ T cells from MCA205 tumors subjected to radiation with or without IL-21. (B) Quantitative analysis of $CD3^+$ T cells, $CD4^+$ T cells, B cells, M-MDSCs, PMN-MDSCs, TAMs, M1-TAMs, DCs, TNF-α^+ $CD8^+$ T cells, $CD69^+$ $CD8^+$ T cells, PD-1^+ $CD8^+$ T cells, Tim-3^+ $CD8^+$ T cells and PD-1^+ Tim-3^+ $CD8^+$ T cells from B16 tumors subjected to radiation with or without IL-21. Data are shown as mean ± SEM (n = 5-7 per group). Statistical analysis was performed using one-way ANOVA followed by Tukey's multiple comparison tests (A-B). ns, $p>0.05$, *$p<0.05$, **$p<0.01$, ***$p<0.001$, ****$p<0.0001$.

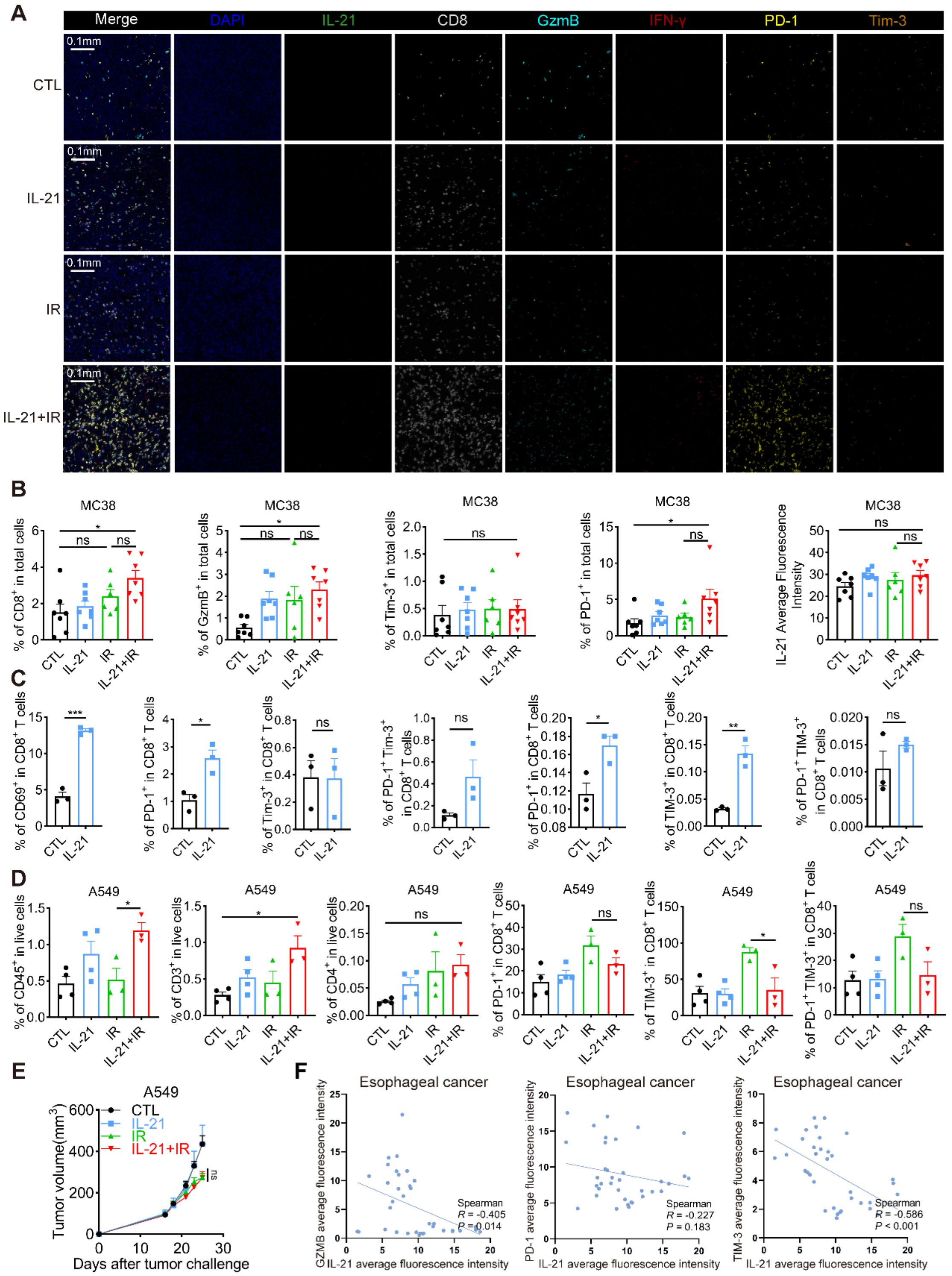

Figure S9. IL-21 directly boosts the activation and cytotoxicity of CD8$^+$ T cells. (A-B) Representative data and quantitative analysis of IL-21, CD8, GzmB, IFN-γ, PD-1 and Tim-3 expression in MC38 tumors subjected to radiation with or without IL-21. (C) Quantitative analysis of CD69$^+$, PD-1$^+$, Tim-3$^+$, PD-1$^+$ Tim-3$^+$ murine

$CD8^+$ T cells (left four panels) and PD-1^+, TIM-3^+, PD-1^+ TIM-3^+ human $CD8^+$ T cells (right three panels) stimulated with anti-CD3 and anti-CD28 in the presence or absence of IL-21. (D) Quantitative analysis of $CD45^+$ immune cells, $CD3^+$ T cells, $CD4^+$ T cells, PD-1^+ $CD8^+$ T cells, TIM-3^+ $CD8^+$ T cells and PD-1^+ TIM-3^+ $CD8^+$ T cells from A549 tumors treated with radiation with or without IL-21 by flow cytometry. (E) Tumor growth of A549 cells in NSG mice subjected to radiation with or without systematic administration of IL-21. (F) Correlation analysis of expression of IL-21 and GZMB, PD-1, TIM-3 in radiotherapy-treated ESCC tissues. Data of Panel B-E are shown as mean ± SEM (n = 3-7 per group) Statistical analysis was performed with one-way ANOVA followed by Tukey's multiple comparison test (B, D) and unpaired two-tailed Student's T test (C), and two-way ANOVA with Tukey's multiple comparison test (E). ns, $p>0.05$, *$p<0.05$, **$p<0.01$, ***$p<0.001$.

Table S1. Patient Information of Lung Adenocarcinoma Tissue Microarray.

Point location	Patient number	Organization Type	Status	Survival period (month)	Sex	Age (year)	Pathological type	Tumor type	Pathological grading	Tumor size (cm)
A01/A02	1	Cancer/Para-cancer	1(Death)	66	Female	53	Lung adenocarcinoma	Peripheral type	2	2×2
A03/A04	2	Cancer/Para-cancer	0 (Survival)	95	Male	49	Lung adenocarcinoma	-	2-3	5×3.5
A05/A06	3	Cancer/Para-cancer	1(Death)	31	Male	62	Lung adenocarcinoma	Peripheral type	2	3.5×2.5
A07/A08	4	Cancer/Para-cancer	1(Death)	2	Female	64	Lung adenocarcinoma	Peripheral type	2	3×2.5
B01/B02	5	Cancer/Para-cancer	0 (Survival)	89	Female	48	Lung adenocarcinoma	Peripheral type	2	2×1.5
B03/B04	6	Cancer/Para-cancer	0 (Survival)	90	Female	53	Lung adenocarcinoma	Peripheral type	2-3	2×2
B05/B06	7	Cancer/Para-cancer	1(Death)	14	Male	71	Lung adenocarcinoma	-	2-3	9.5×6
B07/B08	8	Cancer/Para-cancer	1(Death)	24	Male	72	Lung adenocarcinoma	Central type	3	6×6
C01/C02	9	Cancer/Para-cancer	1(Death)	45	Female	66	Lung adenocarcinoma	Peripheral type	1-2	3×2
C03/C04	10	Cancer/Para-cancer	1(Death)	70	Female	66	Lung adenocarcinoma	Peripheral type	1-2	3×2.5
C05/C06	11	Cancer/Para-cancer	1(Death)	48	Male	49	Lung adenocarcinoma	Central type	2	2.8×2.5
C07/C08	12	Cancer/Para-cancer	1(Death)	34	Female	67	Lung adenocarcinoma	-	2	3×2

D01/D02	13	Cancer/Para-cancer	1(Death)	14	Male	67	Lung adenocarcinoma	Peripheral type	2	3×2.5
D03/D04	14	Cancer/Para-cancer	1(Death)	22	Female	54	Lung adenocarcinoma	Central type	2	4×3；1×0.5
C05/C06	15	Cancer/Para-cancer	1(Death)	60	Female	49	Lung adenocarcinoma	Peripheral type	2	2.5×2
D07/D08	16	Cancer/Para-cancer	1(Death)	63	Female	64	Lung adenocarcinoma	Peripheral type	2	3×2
E01/E02	17	Cancer/Para-cancer	1(Death)	25	Male	61	Lung adenocarcinoma	Central type	2-3	4.5×3
E03/E04	18	Cancer/Para-cancer	1(Death)	36	Female	67	Lung adenocarcinoma	Peripheral type	2	1.5×1
E05/E06	19	Cancer/Para-cancer	0 (Survival)	84	Male	37	Lung adenocarcinoma	Central type	2	2.5×2.5×2
E07/E08	20	Cancer/Para-cancer	1(Death)	36	Male	44	Lung adenocarcinoma	Peripheral type	2	2×1.5
F01/F02	21	Cancer/Para-cancer	0 (Survival)	55	Female	56	Lung adenocarcinoma	Peripheral type	2	2×1.5
F03/F04	22	Cancer/Para-cancer	1(Death)	36	Female	46	Lung adenocarcinoma	Central type	3	4.5×3.5
F05/F06	23	Cancer/Para-cancer	1(Death)	41	Male	53	Lung adenocarcinoma	Central type	2	3×2
F07/F08	24	Cancer/Para-cancer	0 (Survival)	83	Male	47	Lung adenocarcinoma	Central type	2	6×4
G01/G02	25	Cancer/Para-cancer	1(Death)	25	Female	56	Lung adenocarcinoma	Central type	2-3	3×2
G03/G04	26	Cancer/Para-cancer	1(Death)	20	Female	49	Lung adenocarcinoma	Peripheral type	2	6×5

G05/G06	27	Cancer/Para-cancer	1(Death)	25	Male	55	Lung adenocarcinoma	Peripheral type	3	7×6×1.5
G07/G08	28	Cancer/Para-cancer	1(Death)	30	Male	48	Lung adenocarcinoma	Peripheral type	2-3	5×3
H01/H02	29	Cancer/Para-cancer	1(Death)	35	Male	67	Lung adenocarcinoma	Central type	3	2.5×2
H03/H04	30	Cancer/Para-cancer	1(Death)	21	Female	60	Lung adenocarcinoma	Peripheral type	1	2.5×2
H05/H06	31	Cancer/Para-cancer	1(Death)	13	Female	68	Lung adenocarcinoma	Peripheral type	2	2.5×2.5
H07/H08	32	Cancer/Para-cancer	0 (Survival)	43	Male	50	Lung adenocarcinoma	Peripheral type	2	2.3x2
I01/I02	33	Cancer/Para-cancer	1(Death)	41	Male	43	Lung adenocarcinoma	Peripheral type	2	5×3
I03/I04	34	Cancer/Para-cancer	0 (Survival)	79	Female	59	Lung adenocarcinoma	Peripheral type	2	2×2
I05/I06	35	Cancer/Para-cancer	1(Death)	25	Female	40	Lung adenocarcinoma	Peripheral type	2	2.5×3
I07/I08	36	Cancer/Para-cancer	1(Death)	28	Female	56	Lung adenocarcinoma	Peripheral type	2-3	3.5×2.5
J01/J02	37	Cancer/Para-cancer	1(Death)	17	Male	70	Lung adenocarcinoma	Peripheral type	3	6×4
J03/J04	38	Cancer/Para-cancer	1(Death)	65	Female	57	Lung adenocarcinoma	Peripheral type	2	1.4×2.5
J05/J06	39	Cancer/Para-cancer	1(Death)	22	Female	67	Lung adenocarcinoma	Peripheral type	2	6×4
J07/J08	40	Cancer/Para-cancer	0 (Survival)	79	Male	50	Lung adenocarcinoma	Peripheral type	2	2.5×2

K01/K02	41	Cancer/Para-cancer	0 (Survival)	43	Male	67	Lung adenocarcinoma	Peripheral type	2	3.5×3
K03/K04	42	Cancer/Para-cancer	1(Death)	27	Male	71	Lung adenocarcinoma	Central type	2	4×3×3
K05/K06	43	Cancer/Para-cancer	1(Death)	18	Male	58	Lung adenocarcinoma	Peripheral type	2-3	4.5×3.5×3
K07/K08	44	Cancer/Para-cancer	1(Death)	33	Female	44	Lung adenocarcinoma	Peripheral type	2	2×1.5×1
L01/L02	45	Cancer/Para-cancer	1(Death)	24	Male	69	Lung adenocarcinoma	Peripheral type	2-3	2.5×2.4×2.5
L03/L04	46	Cancer/Para-cancer	1(Death)	84	Female	62	Lung adenocarcinoma	Peripheral type	2	2.5
L05/L06	47	Cancer/Para-cancer	0 (Survival)	89	Male	52	Lung adenocarcinoma	Peripheral type	2	2
L07/L08	48	Cancer/Para-cancer	1(Death)	26	Male	74	Lung adenocarcinoma	-	2	17×12
M01/M02	49	Cancer/Para-cancer	1(Death)	18	Male	51	Lung adenocarcinoma	-	3	3×2
M03/M04	50	Cancer/Para-cancer	1(Death)	51	Female	51	Lung adenocarcinoma	Peripheral type	2	3×2
M05/M06	51	Cancer/Para-cancer	1(Death)	17	Female	58	Lung adenocarcinoma	-	2	4×3
M07/M08	52	Cancer/Para-cancer	0 (Survival)	84	Female	62	Lung adenocarcinoma	-	1	3×2
N01/N02	53	Cancer/Para-cancer	1(Death)	13	Female	64	Lung adenocarcinoma	Peripheral type	2	4.5×4
N03/N04	54	Cancer/Para-cancer	0 (Survival)	82	Female	53	Lung adenocarcinoma	Peripheral type	2	2×2

N05/N06	55	Cancer/Para-cancer	1(Death)	50	Male	60	Lung adenocarcinoma	Peripheral type	2	3×2
N07/N08	56	Cancer/Para-cancer	0 (Survival)	80	Male	59	Lung adenocarcinoma	Peripheral type	2	3×2
O01/O02	57	Cancer/Para-cancer	1(Death)	29	Male	60	Lung adenocarcinoma	Peripheral type	2-3	4×3.5
O03/O04	58	Cancer/Para-cancer	0 (Survival)	77	Female	50	Lung adenocarcinoma	Peripheral type	2	3×3
O05/O06	59	Cancer/Para-cancer	1(Death)	49	Female	69	Lung adenocarcinoma	Peripheral type	2	2.5×2.5
O07/O08	60	Cancer/Para-cancer	1(Death)	52	Female	60	Lung adenocarcinoma	Peripheral type	3	1.5×3

Table S2. Clinical information of patients with esophageal squamous cell carcinoma administrated with neoadjuvant radiotherapy.

Patient number	Sex	Age (year)	Status	Survival period (month)	Preoperative pathology	Treatment method
1	Male	59	0(Survival)	65	Esophageal Squamous Cell Carcinoma	Neoadjuvant radiotherapy + radical esophagectomy for esophageal cancer
2	Male	70	0(Survival)	63	Esophageal Squamous Cell Carcinoma	Neoadjuvant radiotherapy + radical esophagectomy for esophageal cancer
3	Male	48	0(Survival)	61	Esophageal Squamous Cell Carcinoma	Neoadjuvant radiotherapy + radical esophagectomy for esophageal cancer
4	Female	72	0(Survival)	60	Esophageal Squamous Cell Carcinoma	Neoadjuvant radiotherapy + radical esophagectomy for esophageal cancer
5	Male	54	0(Survival)	60	Esophageal Squamous Cell Carcinoma	Neoadjuvant radiotherapy + radical esophagectomy for esophageal cancer
6	Male	56	0(Survival)	60	Esophageal Squamous Cell Carcinoma	Neoadjuvant radiotherapy + radical esophagectomy for esophageal cancer
7	Male	60	0(Survival)	59	Esophageal Squamous Cell Carcinoma	Neoadjuvant radiotherapy + radical esophagectomy for esophageal cancer
8	Female	68	0(Survival)	58	Esophageal Squamous Cell Carcinoma	Neoadjuvant radiotherapy + radical esophagectomy for esophageal cancer
9	Female	58	0(Survival)	55	Esophageal Squamous Cell Carcinoma	Neoadjuvant radiotherapy + radical esophagectomy for esophageal cancer
10	Male	55	1(Death)	20	Esophageal Squamous Cell Carcinoma	Neoadjuvant radiotherapy + radical esophagectomy for esophageal cancer
11	Male	71	0(Survival)	54	Esophageal Squamous Cell Carcinoma	Neoadjuvant radiotherapy + radical esophagectomy for esophageal cancer
12	Male	61	1(Death)	41	Esophageal Squamous Cell Carcinoma	Neoadjuvant radiotherapy + radical esophagectomy for esophageal cancer
13	Male	67	1(Death)	15	Esophageal Squamous Cell Carcinoma	Neoadjuvant radiotherapy + radical esophagectomy for esophageal cancer

14	Male	61	0(Survival)	52	Esophageal Squamous Cell Carcinoma	Neoadjuvant radiotherapy + radical esophagectomy for esophageal cancer
15	Male	55	0(Survival)	51	Esophageal Squamous Cell Carcinoma	Neoadjuvant radiotherapy + radical esophagectomy for esophageal cancer
16	Male	76	0(Survival)	51	Esophageal Squamous Cell Carcinoma	Neoadjuvant radiotherapy + radical esophagectomy for esophageal cancer
17	Male	59	0(Survival)	50	Esophageal Squamous Cell Carcinoma	Neoadjuvant radiotherapy + radical esophagectomy for esophageal cancer
18	Female	67	1(Death)	10	Esophageal Squamous Cell Carcinoma	Neoadjuvant radiotherapy + radical esophagectomy for esophageal cancer
19	Male	58	1(Death)	22	Esophageal Squamous Cell Carcinoma	Neoadjuvant radiotherapy + radical esophagectomy for esophageal cancer
20	Male	65	1(Death)	25	Esophageal Squamous Cell Carcinoma	Neoadjuvant radiotherapy + radical esophagectomy for esophageal cancer
21	Male	66	0(Survival)	48	Esophageal Squamous Cell Carcinoma	Neoadjuvant radiotherapy + radical esophagectomy for esophageal cancer
22	Female	54	1(Death)	10	Esophageal Squamous Cell Carcinoma	Neoadjuvant radiotherapy + radical esophagectomy for esophageal cancer
23	Male	68	0(Survival)	48	Esophageal Squamous Cell Carcinoma	Neoadjuvant radiotherapy + radical esophagectomy for esophageal cancer
24	Male	56	0(Survival)	48	Esophageal Squamous Cell Carcinoma	Neoadjuvant radiotherapy + radical esophagectomy for esophageal cancer
25	Male	69	0(Survival)	47	Esophageal Squamous Cell Carcinoma	Neoadjuvant radiotherapy + radical esophagectomy for esophageal cancer
26	Male	72	0(Survival)	47	Esophageal Squamous Cell Carcinoma	Neoadjuvant radiotherapy + radical esophagectomy for esophageal cancer
27	Male	63	0(Survival)	47	Esophageal Squamous Cell Carcinoma	Neoadjuvant radiotherapy + radical esophagectomy for esophageal cancer
28	Female	70	0(Survival)	45	Esophageal Squamous Cell Carcinoma	Neoadjuvant radiotherapy + radical esophagectomy for esophageal cancer

29	Male	57	0(Survival)	45	Esophageal Squamous Cell Carcinoma	Neoadjuvant radiotherapy + radical esophagectomy for esophageal cancer
